# Nanocatalytic Immunotherapy Targeting Tumor‐Resident Intracellular Bacteria for Triple‐Negative Breast Cancer Treatment and Metastasis Suppression

**DOI:** 10.1002/advs.78559

**Published:** 2026-10-31

**Authors:** Xiaojing Leng, Tao Jiang, Zhao Guo, Zhimin Zhang, Yihan Chen, Pan Li, Min Ge, Han Lin, Liang Zhang

**Affiliations:** ^1^ Department of Ultrasound The First Affiliated Hospital of Chongqing Medical University Chongqing People's Republic of China; ^2^ Department of Anaesthesiology Chongqing Traditional Chinese Medicine Hospital Chongqing People's Republic of China; ^3^ Department of Orthopedic Surgery Shanghai Institute of Microsurgery on Extremities Shanghai Sixth People's Hospital Affiliated to Shanghai Jiao Tong University School of Medicine Shanghai People's Republic of China; ^4^ Shanghai Institute of Ceramics Chinese Academy of Sciences Research Unit of Nanocatalytic Medicine in Specific Therapy for Serious Disease Chinese Academy of Medical Sciences Shanghai People's Republic of China; ^5^ Department of Electrical Engineering City University of Hong Kong Hong Kong SAR People's Republic of China

**Keywords:** antitumor immunity, nanosheets, triple‐negative breast cancer, tumor‐resident intracellular bacteria, ultrasoundresponsive

## Abstract

Tumor‐resident intracellular bacteria (TRIB) are prevalent within triple‐negative breast cancer (TNBC). As integral components of the tumor pathological microenvironment, TRIB accelerate TNBC development by regulating cell proliferation, metabolism and immunosuppression. They also enhance the resistance of circulating tumor cells to mechanical stress through cytoskeletal rearrangement, boosting tumor metastasis and hindering clinical therapy. This work develops an advanced TRIB‐targeted therapeutic strategy to block TNBC progression by eliminating tumor cells and intracellular bacteria, remodeling tumor immunity and suppressing metastatic colonization. We fabricate cancer cell membrane‐modified narrow‐bandgap FeSe_2_ nanosheets for enhanced ultrasound‐triggered reactive oxygen species production and image‐guided precise treatment. Such nanoplatforms utilize endogenous H_2_O_2_ and GSH to amplify chemodynamic effects, induce severe oxidative stress and ferroptosis in tumor cells. Combined damage‐associated and pathogen‐associated molecular patterns efficiently activate dendritic cells and initiate robust antigen‐specific T‐cell immune responses. This study provides an alternative and promising strategy for the treatment of clinically metastatic breast cancer through intratumoral microbiota intervention.

## Graphical abstract

Bacteria residing within tumor cells contribute to breast cancer progression, but few therapies target their elimination. FeSe_2_ nanosheets coated with cancer cell membranes accumulate in tumors and generate reactive oxygen species under ultrasound, killing tumor cells and their harbored bacteria at the same time. This dual effect stimulates antitumor immunity and markedly inhibits tumor growth and lung metastasis.

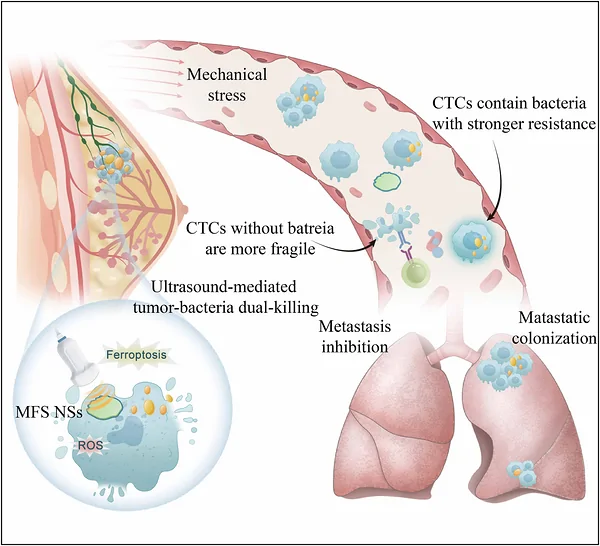

## 1 Introduction

Triple‐negative breast cancer (TNBC), the most aggressive subtype of breast cancer, is characterized by high recurrence, metastasis, and poor prognosis, representing a significant clinical challenge [1]. Research indicates that metastasis contributes to the low 5‐year survival rate in cancer patients [2, 3]. Previous studies, operating under the assumption of a sterile tumor microenvironment, have primarily focused on the interactions between tumor cells and host cells during metastasis [4, 5]. The accumulating evidence is increasingly recognizing the existence of a microbial community with low biomass but functionally significant within the tumor tissue, encompassing various cancer types such as breast, pancreatic, and lung carcinomas, among others, challenging the previous assumption of these tissues as sterile environments [6, 7, 8, 9]. Notably, tumor‐resident intracellular bacteria (TRIB) are demonstrated to have direct influences on the proliferation, metabolic activity, and invasive potential of tumor cells, while also potentiating immunosuppressive mechanisms and promoting distant metastasis of tumors [10, 11, 12, 13]. Research indicates that the presence of bacteria within circulating tumor cells (CTCs) leads to a substantial reorganization of the actin cytoskeleton, which markedly enhances the resilience of these cells during the process of metastasis [13, 14]. This adaptation endows CTCs with enhanced resilience against the mechanical challenges inherent in hematogenous dissemination, thereby ensuring their protection and potentiating their ability to achieve successful metastatic colonization. Given the pivotal role of TRIB in the progression of cancer, the conceptualization of a therapeutic modality that converges on the elimination of both malignant cells and their intracellular bacteria may emerge as an innovative and efficacious strategy for anticancer interventions.

Reactive oxygen species (ROS) constitute a class of oxygen‐derived molecules with significant chemical reactivity, capable of oxidizing crucial biomacromolecules such as DNA, proteins, and lipids, ultimately leading to cellular damage and apoptosis [15, 16, 17]. This oxidative damage serves as a pivotal mechanism for eradicating tumor cells and bacterial populations, forming the foundational framework for anti‐neoplastic and antibacterial therapeutic strategies [18, 19]. As an emerging strategy for ROS generation, sonodynamic therapy (SDT) has gained significant attention in the medical field in recent years, attributed to its substantial advantages of non‐invasiveness, low toxicity, good penetration, spatiotemporal controllability and repeatability [20, 21, 22]. Inorganic nanomaterials, have demonstrated great potential as emerging sonosensitizers [23, 24]. US‐induced separation of electron (e^−^) and hole (h^+^) pairs within these nanomaterials, coupled with the released energy, facilitates redox reactions with O_2_ and H_2_O, leading to the generation of abundant cytotoxic ROS [25]. Therefore, further expanding the varieties of inorganic sensitizers, optimizing their structural design, and enhancing their targeted accumulation at tumor sites, represents a pivotal direction for advancing the translation of SDT from bench to bedside. Empirical evidence suggests that the efficiency of ROS generation in SDT is determined by the bandgap energy separating the valence and conduction bands of the employed sonosensitizers. In comparison to the classical sonosensitizer TiO_2_, which possesses a wide band gap (3.2 eV) leading to rapid electron‐hole recombination and limited ROS generation [26], SnS with its narrower band gap (1.07–1.81 eV) exhibits a substantially higher ROS production efficiency when exposed to ultrasonic irradiation [27]. Therefore, optimizing sonosensitizer design to achieve narrow bandgaps represents a crucial strategy for promoting the efficient generation of ROS in SDT. Encouragingly, the lethal effects induced by ROS results in the release of damage‐associated molecular patterns (DAMPs) from tumor cells and pathogen‐associated molecular patterns (PAMPs) from TRIB, respectively [28, 29]. These molecular patterns have been demonstrated to possess the potential to induce enhanced immune responses, providing a robust foundation for the suppression of tumor proliferation and prevention of distant metastasis [30, 31, 32].

Herein, we innovatively present a tumor‐bacteria dual‐killing immunotherapy based on cell membrane‐engineered FeSe_2_ nanosheets (MFS NSs), which were engineered from a narrower bandgap of the developed FeSe_2_ nanosheets (FS NSs) allows a more effortless separation of sono‐triggered electron‐hole pairs for ROS generation, and were subsequently surface‐modified with 4T1 cancer cell membranes (CCMs) to facilitate imaging‐guided precision therapy [33, 34, 35]. Following the imaging‐based monitoring revealed a high concentration accumulation of nanosheets within the tumor tissues, ultrasound (US) irradiation was applied to induce the generation of substantial amounts of ROS, leading to lethal attacks on both tumor cells and intracellular bacteria.

Meanwhile, MFS initiated a tumor microenvironment‐activated chemodynamic therapy (CDT) process, which induced a self‐propagating cycle of ferrous ion regeneration via interactions with intracellular H_2_O_2_ and glutathione (GSH) to amplify the intracellular oxidative burden and initiate ferroptosis [36]. The elimination of TRIB significantly influences the attenuation of immunosuppressive mechanisms and constitutes a pivotal strategy for inhibiting the process of distant metastasis by reducing the resilience of CTCs [10, 13]. Notably, the DAMPs released from dying tumor cells and the PAMPs secreted from the destroyed TRIB, can induce a potent in situ and systemic anti‐tumor immune response, culminating in the comprehensive inhibition of the primary tumor and marked reduction of distant metastasis (Scheme 1). Furthermore, the MFS NSs demonstrate exceptional biocompatibility and facilitate efficient renal clearance, devoid of appreciable adverse toxicological effects in the treated animals. In summary, the present tumor—intracellular bacteria combined therapeutic has demonstrated a synergistic immunotherapeutic efficacy against tumor proliferation, bacterial colonization, and metastatic spread. This strategy of targeting the tumor cell microbiome as an intervention target provides a novel paradigm and perspective for the clinical treatment of breast cancer and other metastatic cancers.

**SCHEME 1:**
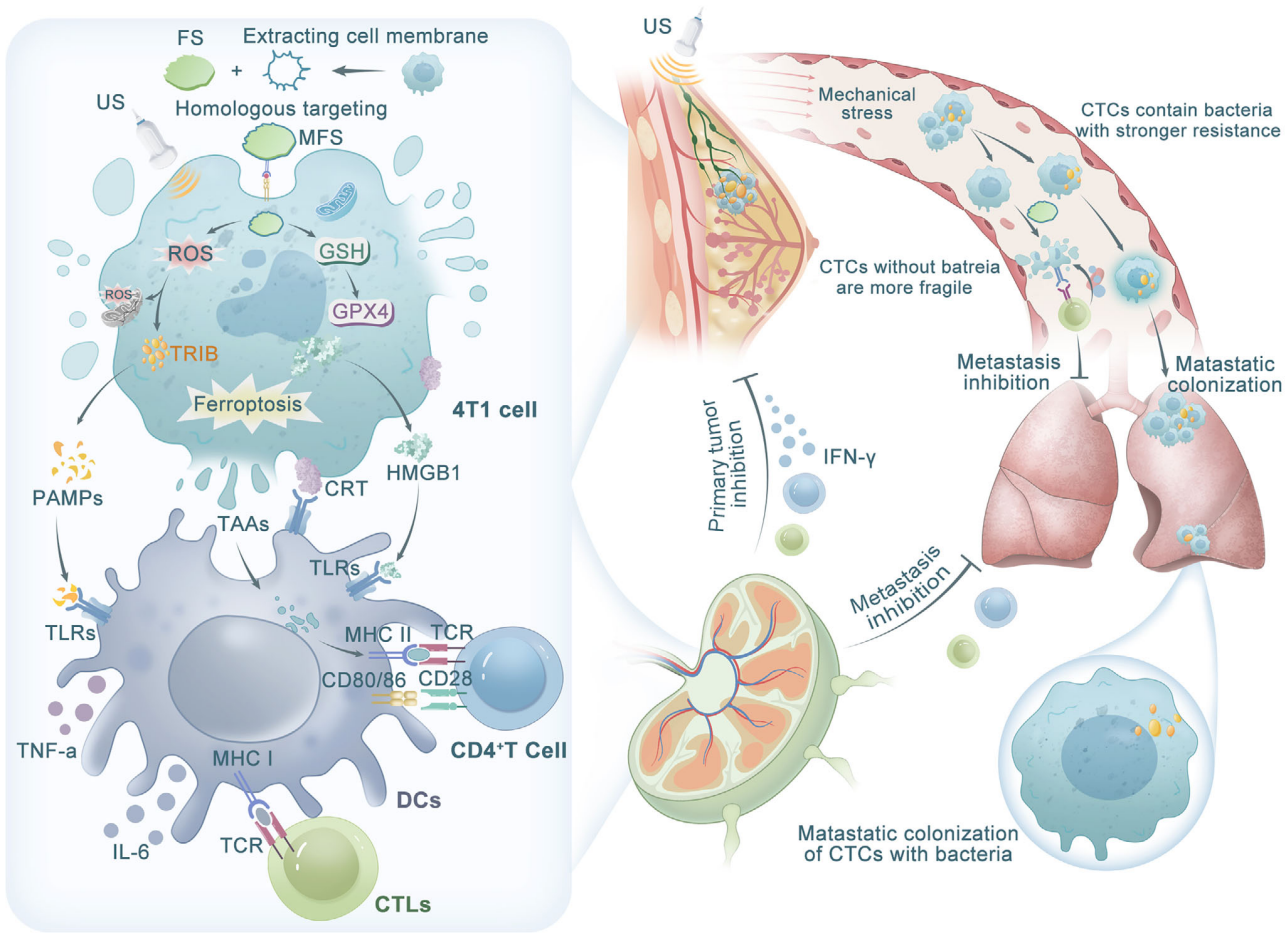
The schematic illustrates an emerging anti‐tumor immunotherapeutic strategy that utilizes the combined application of SDT and CDT to concurrently target and eliminate tumor cells and their intracellular bacteria, thereby eliciting potent immune responses that inhibit tumor growth and metastasis. The developed FeSe_2_ nanosheets, characterized by their tumor‐targeting efficacy and the enhanced ROS generation ability under US stimulation, promote ferroptosis and oxidative stress. Subsequently, the released DAMPs and PAMPs contribute to the induction of a robust immune response. The elimination of bacteria within CTCs can reduce their resistance to mechanical stresses during the metastatic process, thereby compromising their potential for establishing metastatic colonization.

## 2 Results

### 2.1 Characterization of Bacterial Communities in Cancerous Tissues of TNBC Patients

We characterized the bacterial communities by performing five‐region 16S ribosomal RNA (5R 16S rRNA) gene sequencing on matched pairs of cancerous and para‐cancerous tissue samples obtained from TNBC patients (Figure 1a). Principal coordinate analysis (PCoA) revealed significant differences in the microbial community structure between the operating room air control, the cancerous and para‐cancerous tissue samples (Figure 1b). The results preliminarily indicate that the bacterial community composition in the cancerous and para‐cancerous tissues of TNBC patients is significantly different from that of the control group, and there may also be heterogeneity in the microbial composition between the cancerous and para‐cancerous tissues.

**FIGURE 1:**
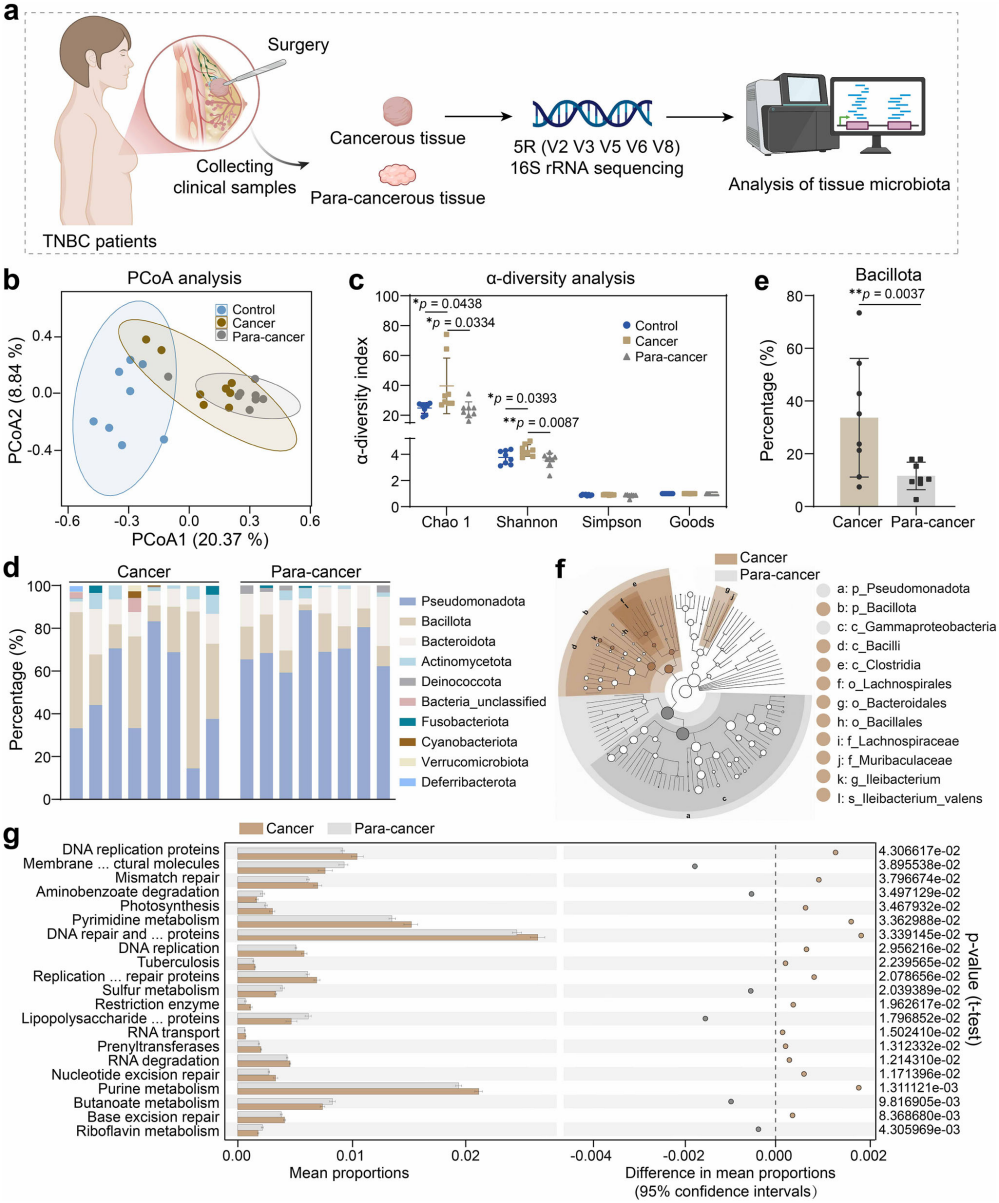
5R 16S rRNA gene sequencing analysis of cancerous and para‐cancerous tissues of TNBC patients. (a) Schematic diagram showing the workflow for 5R 16S rRNA gene sequencing of tissue‐resident microbiota in cancerous and para‐cancerous tissues (*n* = 8). (b) Differences in microbial communities were compared using PcoA analysis. (c) Alpha diversity indices, including Chao, Shannon, Simpson, and Good's coverage, were used to assess microbial community richness and evenness. (d) The bar graph plots each patient's relative abundance of the top 10 enriched phylum taxonomies. (e) Relative abundance differences of Bacillota. (f) LEfSe analysis was used to identify bacterial taxa that were significantly different between groups (LDA score > 3). (g) KEGG pathway analysis of microbial functional differences between cancerous and para‐cancerous tissues predicted by PICRUSt2 and analyzed using STAMP.

Alpha diversity analysis revealed that the Chao1 index for species richness and the Shannon index for species diversity were significantly higher in the cancerous tissues of TNBC patients than in the control and para‐cancerous groups (Figure 1c). However, no significant differences were observed among the three groups in the Simpson index, which reflects species dominance, or in Goods’ coverage, which assesses sequencing depth. This indicates that the bacterial community in TNBC cancerous tissues possesses higher richness and overall diversity, while its dominance structure and sequencing depth are comparable to those of the control and para‐cancerous tissues. This pattern suggests that the additional species recruited into TNBC tissues remain at low relative abundance and do not displace the pre‐existing dominant taxa, indicating a richness‐driven dysbiosis characterized by expansion of the rare biosphere rather than overgrowth of a single pathobiont.

Analysis of the top 10 most abundant taxa at the phylum level revealed distinct bacterial community compositions between cancerous and para‐cancerous tissues, with *Bacillota* being significantly enriched in cancerous tissues (Figure 1d,e). Furthermore, the linear discriminant analysis effect size (LEfSe) analysis, with a linear discriminant analysis (LDA) score threshold of 3.0, identified distinct microbial biomarkers between cancerous and para‐cancerous tissues (Figure 1f). At the phylum level, *Pseudomonadota* was significantly enriched in para‐cancerous tissues, whereas *Bacillota* was enriched in cancerous tissues. Further analysis revealed significant differences at lower taxonomic ranks, such as *Bacilli*, *Clostridia*, *Lachnospirales*, *Bacteroidales*, *Lachnospiraceae*, *Muribaculaceae*, *Ileibacterium*, all of which were significantly enriched in cancerous tissues.

To investigate the potential impact of functional differences in the bacterial communities on tumor tissues, we employed Phylogenetic Investigation of Communities by Reconstruction of Unobserved States (PICRUSt) to predict functional gene composition (Figure 1g). The analysis revealed significant disparities in several KEGG pathways between the two groups. Notably, pathways related to DNA replication, DNA repair and recombination proteins, and nucleotide metabolism (pyrimidine and purine metabolism) were significantly enriched in cancerous tissues. These pathways are central to cellular proliferation and DNA synthesis, suggesting that these bacterial communities may contribute to enhanced tumor cell growth and proliferation. These findings indicate that the distinct microbial communities in TNBC cancerous and para‐cancerous tissues possess different functional potentials, with the tumor‐associated microbiota potentially promoting tumor progression by modulating key metabolic and biosynthetic processes.

To further determine the intracellular localization of the bacteria, 16S ribosomal RNA fluorescence in situ hybridization (16S rRNA FISH) was performed on tumor sections from TNBC patients. Discrete 16S‐positive signals were clearly detected within the tumor region, and part of these signals overlapped with or were surrounded by DAPI‐stained tumor cell nuclei, indicating the presence of bacteria within tumor cells (Figure S1a). This finding was further corroborated by biological transmission electron microscopy (Bio‐TEM) analysis, which revealed bacterial structures with cell walls located in the cytosol of tumor cells (Figure S1b). Collectively, these results provide direct visual evidence that the bacteria residing in TNBC cancerous tissues are at least partially localized within tumor cells.

### 2.2 Influence of TRIB on Tumor Cell Migration and Metastatic Colonization

Having identified bacterial communities in TNBC cancerous tissues, we next sought to directly verify whether these TRIB functionally promote malignant phenotypes, a premise that underpins our therapeutic design. To this end, a proof‐of‐principle intracellular infection model was established by inoculating *Staphylococcus aureus (S. aureus)* into 4T1 cells, which served as a controlled and tractable system to mimic intracellular bacterial infection, rather than a direct replication of endogenous TRIB.

On this basis, the impact of intracellular bacteria on the migratory capacity of 4T1 cells was first evaluated. Transwell migration and wound‐healing assays were subsequently performed to compare the migratory capacities of untreated 4T1 cells and *S. aureus*‐harboring 4T1 cells. In the wound‐healing assay, the scratched areas of both groups gradually narrowed over time; notably, *S. aureus*‐harboring 4T1 cells exhibited a markedly faster wound closure, with the scratched region nearly closed at 48 h, whereas a distinct gap remained in the untreated 4T1 group (Figure S2a). Quantitative analysis confirmed that the migration rate of *S. aureus*‐harboring 4T1 cells was approximately 2‐fold higher than that of untreated 4T1 cells (Figure S2b). This promoting effect was further validated by the Transwell migration assay, in which the number of cells traversing the membrane was significantly increased in the *S. aureus*‐harboring group (Figure S2c), rising from approximately 130 to 260 cells (Figure S2d). Collectively, these results indicate that intracellular bacterial colonization substantially promotes the migration of 4T1 cells in vitro, suggesting that TRIB may endow tumor cells with enhanced motility, thereby potentially facilitating tumor progression and metastatic dissemination.

To directly assess the metastasis‐promoting function of TRIB in vivo, we established an experimental lung metastasis model by intravenous injection of 4T1 cells or *S. aureus*‐harboring 4T1 cells into mice, thereby mimicking and evaluating the colonization capacity of CTCs in lung tissue. Compared with the 4T1 group, mice injected with S. aureus‐harboring 4T1 cells developed approximately 3‐fold more lung metastatic nodules, providing direct functional evidence that intracellular bacteria enhance the mechanical tolerance of CTCs in the bloodstream and their subsequent tissue colonization ability (Figure S3).

### 2.3 Synthesis and Characterizations of FS NSs

The FS NSs were synthesized via the hydrothermal method and modified with amino‐polyethylene glycol [37]. Moreover, PEG modification markedly improved the colloidal dispersity of the FS NSs. As shown in Figure S4a, after standing for 4 h, the unmodified FS dispersion exhibited obvious sedimentation, whereas the FS‐PEG dispersion remained stable and homogeneous. Consistently, the PEGylated FS NSs showed a much lower polydispersity index (PDI) of 0.167 ± 0.036 than the unmodified FS NSs of 0.492 ± 0.035 (Figure S4b), confirming that PEG decoration effectively suppressed the aggregation of FS NSs in aqueous dispersion. Scanning electron microscopy and TEM images revealed that the as‐synthesized FS NSs possess a two‐dimensional layered structure, while the corresponding ball‐and‐stick model further elucidates their three‐dimensional atomic framework, confirming excellent crystallinity and structural stability (Figure 2a,b). The elemental composition and distribution of FS NSs were investigated using energy‐dispersive x‐ray spectrometry, where the elemental mapping exhibited a uniform distribution of Fe and Se (Figure 2c). The x‐ray powder diffraction pattern obtained for the FS NSs revealed that it possesses a crystal structure identical to that of orthorhombic FeSe_2_, as reported in the standard JCPDS Card (PDF#21‐0423) (Figure 2d). The Raman spectrum of the synthesized FS NSs revealed prominent peaks at 215.65 cm^−1^ and 280.64 cm^−1^, which are primarily attributed to the stretching modes of the Se_2_ units within the material (Figure 2e). These spectral features are attributable to the vibrational modes of the Se‐Se bonds, encompassing both translational and stretching motions, or potentially a superposition of these vibrational modes. The peak observed at 380.81 cm^−^^1^ was ascribed to a quantitative effect, arising from the abundance of surface‐active reaction sites facilitated by the two‐dimensional nanosheet structure. These results provided compelling evidence for the successful synthesis of the FeSe_2_ structure. Moreover, the surface composition and chemical status of the FS NSs were further evaluated by x‐ray Photoelectron Spectroscopy (XPS) (Figure 2f–h). The high‐resolution Fe 2p spectrum revealed that the predominant peaks at 710.38 eV and 723.39 eV were ascribed to Fe 2p_3/2_ and Fe 2p_1/2_, respectively, with a spin‐energy separation of approximately 13.01 eV, indicative of the characteristics of Fe^2+^ of FS NSs. Simultaneously, characteristic features corresponding to a minor proportion of Fe^3+^ were discernible at absorption peaks located at 713.16 eV and 726.23 eV. Furthermore, a detailed analysis of the Se 3d spectrum revealed that the peaks located at 54.90 eV and 55.87 eV, as well as those at 55.58 eV and 56.62 eV, match well within a certain tolerance with the Se 3d_5/2_ and Se 3d_3/2_ spin‐orbit split peaks, respectively, which are characteristic XPS peaks of the Se element [37, 38]. And minor surface oxidation state was identified based on the broad peak at 58.71 eV, which was attributed to the high surface reactivity of the nanosheet structure.

**FIGURE 2:**
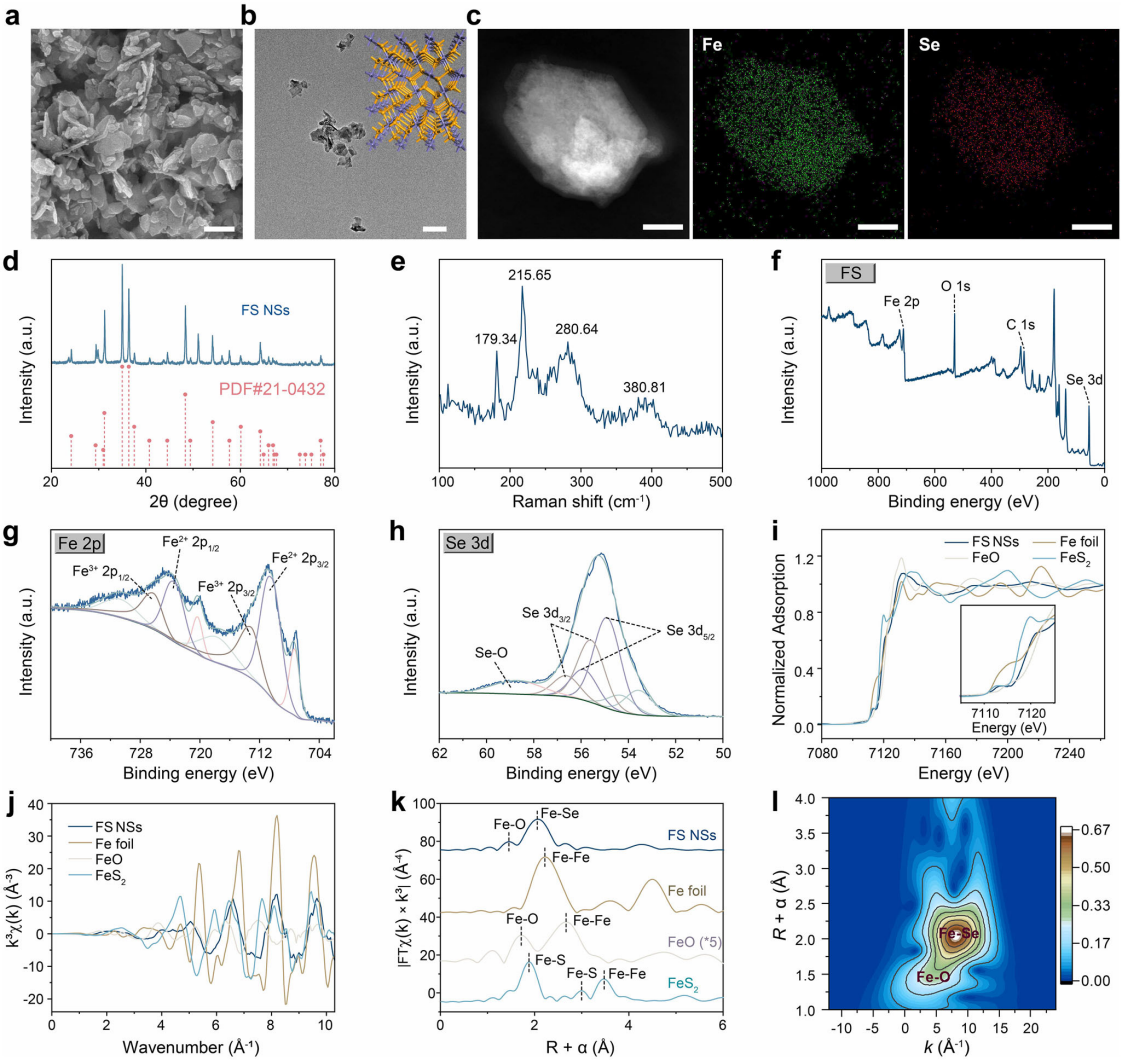
Structural characterizations of FS NSs. (a) Representative scanning electron microscopy image of FS NSs (scale bar: 200 nm). (b) Representative TEM image of FS NSs and the corresponding ball‐and‐stick representation of their crystal structure (scale bar: 200 nm). (c) The elemental mapping of Fe and Se of FS NSs (scale bar: 50 nm). (d) X‐ray powder diffraction pattern and (e) Raman spectra of FS NSs.XPS data showing the (f) survey spectrum, (g) Fe 2p spectrum, and (h) Se 3d spectrum of FS NSs. (i) Normalized XANES spectra of the Fe K‐edge for FS NSs, Fe foil, FeO, and FeS_2_. (j) k_3_‐weighted EXAFS spectra. (k) Fourier‐transform Fe k‐edge EXAFS spectra of FS NSs, Fe foil, FeO, and FeS_2_. (l) Wavelet transform images for the EXAFS signals of FS NSs.

To determine the electronic configuration and coordination environment of Fe in FS NSs, we analyzed the x‐ray absorption near‐edge structure (XANES) spectra at the Fe k‐edge [39]. The results indicate that the absorption edge of FS NSs is similar to that of FeO and FeS_2_, confirming the dominance of the Fe^2+^ oxidation state (Figure 2i). The k_3_‐weighted extended x‐ray absorption fine structure (EXAFS) in k‐space and the corresponding Fourier transform spectra in R‐space were further employed to characterize the coordination environment around the Fe sites [40] (Figure 2j,k). The corresponding Fourier transform extended x‐ray absorption fine structure spectrum shows a prominent peak at approximately 2.06 Å, attributed to Fe–Se coordination [41]. Furthermore, high‐resolution wavelet transformation (WT) analysis was performed on Fe K‐edge oscillations to resolve the overlapping Fe–Se and Fe–O coordination in k‐R space, which confirms the coexistence of Fe‐centered Fe‐Se dominant structure and peripheral Fe‐O bonding [42], consistent with the EXAFS analysis (Figure 2l).

### 2.4 ROS Catalytic Performance and Mechanism of FS NSs

Subsequently, we further verified the US‐triggered ROS generation performance of FS NSs. The optical absorption spectrum of FS NSs was analyzed using UV–vis spectroscopy (Figure 3a). Utilizing the Tauc plot derived from the Kubelka–Munk function, the optical bandgap of the FS NSs was determined to be 1.12 eV. Owing to the narrow bandgap of FS NSs, the separation of electrons (e^−^) and holes (h^+^) becomes readily achievable under US irritation [33, 43]. To further elucidate the electronic structure of FS NSs, density functional theory calculations were employed to compute the density of states (DOS). The DOS plot delineates the distribution of electronic states across different energy levels, highlighting the contribution of various atomic orbitals to the overall electronic structure (Figure 3b). Notably, the DOS analysis identified a calculated band gap of approximately 1.13 eV, similar to the experimentally determined band gap and underscoring the consistency between experimental data and theoretical predictions. The narrow band gap of FS NSs, which promotes efficient separation of US‐triggered electron‐hole pairs to generate substantial ROS, endows them with the potential to serve as highly effective sonosensitizers. Upon exposure to external ultrasonic irradiation, the efficiently separated electron‐hole pairs of FS NSs engage in redox reactions with adsorbed oxygen molecules, culminating in the generation of singlet molecular oxygen (^1^O_2_). Simultaneously, Fenton reaction between iron species allows a cyclical reaction of the Fenton reaction and the consecutive consumption of GSH (Figure 3c). Next, to investigate the sonodynamic performance, 1,3‐diphenylisobenzofuran (DPBF) was used to detect the generation of ^1^O_2_. In the UV–Vis absorption spectra, a time‐dependent decrease was observed in the absorbance peak of DPBF at 420 nm during exposure to US, demonstrating that ^1^O_2_ generated by US‐triggered FS NSs oxidized DPBF into colorless 1,2‐dibenzoylbenzene over time (Figure 3d,e). However, the absorbance of DPBF showed a negligible decrease under ultrasonic irradiation without the introduction of FS NSs (Figure S5). And the electron paramagnetic resonance (EPR) spectroscopy was employed to investigate the generation of ·OH. An ESR spectrum in the FS + H_2_O_2_ group exhibited a characteristic quartet signal with an intensity ratio of 1:2:2:1, providing definitive evidence for the formation of ·OH via the Fenton reaction mechanism (Figure 3f). Concurrently, the production of ·OH was further substantiated through the utilization of 3,3',5,5'‐Tetramethylbenzidine and 4,4'‐Bi‐2,6‐xylidine (TMB) as indicators. The intensification of TMB coloration demonstrates a concentration‐dependent trend with increasing concentrations of FS NSs. Concurrently, an enhancement of peak intensities at 370 and 652 nm in the UV‐Vis absorption spectrum is observed, which serves as indicative of a substantial production of ·OH (Figure 3g,h). These results indicates that FS NSs can effectively generate a considerable quantity of ROS through the pathways of SDT and CDT, demonstrating its profound potential for the therapeutic applications. The GSH depletion was verified using GSH content detection kit. Within a solution designed to replicate the intracellular pH microenvironment, the depletion of GSH exhibited a concentration‐dependent response relative to the concentration of FS NSs (Figure 3i).

**FIGURE 3:**
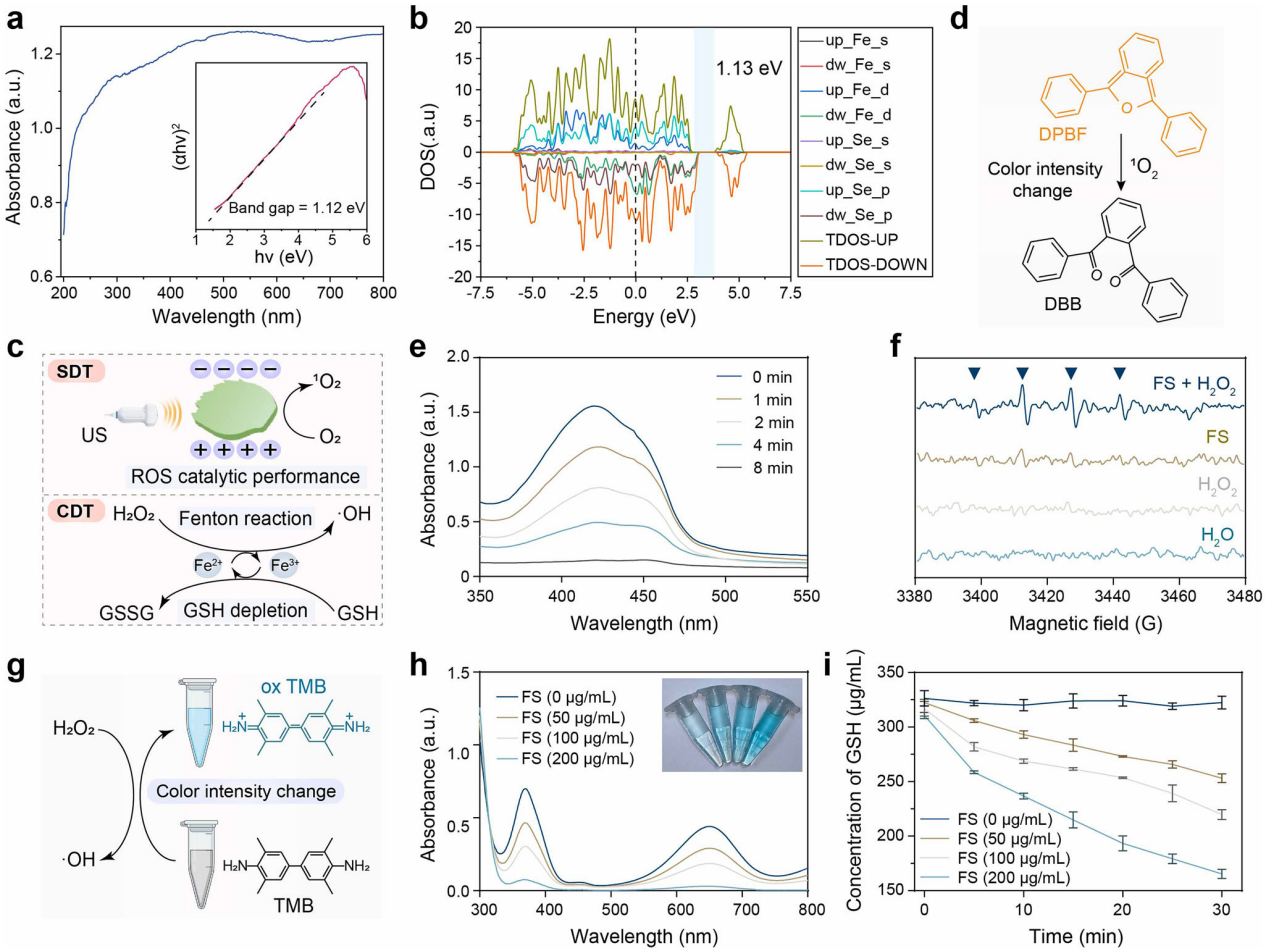
ROS catalytic performance and mechanism of FS NSs. (a) UV–vis diffuse reflectance spectra and tauc plots of FS NSs. (b) Calculated DOS of FS NSs. (c) Schematic illustration of sonodynamic and chemodynamic properties of FS NSs. (d) Schematic diagram of the structural and color intensity changes of DPBF under the action of ^1^O_2_. (e) Time‐dependent oxidation of DPBF by FS‐based ^1^O_2_ generation under US irradiation. (f) ESR spectra demonstrating ·OH generation by Fenton reaction. (g) The illustration depicting the oxidation mechanism of TMB by ·OH, resulting in a colorimetric transition to blue. (h) The colorimetric response and UV–vis absorption spectra of TMB exhibit concentration‐dependent ·OH generation by FS NSs. (i) Concentration‐dependent GSH depletion by FS NSs.

### 2.5 Synthesis, Characterization, and Biocompatibility of MFS NSs

The attainment of a high‐concentration accumulation of nanomaterials within target cells constitutes a pivotal foundation for the deployment of efficacious therapeutic interventions [44]. Functionalized by adhesion proteins derived from cancer cells on the surface, the CCMs‐coated FS NSs (MFS) are expected to demonstrate precise and specific homologous targeting abilities [45]. The TEM image of the MFS NSs clearly revealed the successful coating of CCMs on the surface of the nanosheets (Figure 4a). The elemental mapping images demonstrated the additional distribution of major elements (N, P) of CCMs within MFS NSs (Figure 4b). Furthermore, the sodium dodecyl sulfate‐polyacrylamide gel electrophoresis (SDS‐PAGE) proteomic profiling depicted in Figure 4c indicated that the MFS NSs exhibit distinct protein bands consistent with those derived from the 4T1 CCMs, thereby providing definitive confirmation of the effective membrane coating process. Remarkably, the MFS NSs exhibit a moderately negative surface charge (Figure 4d), which provides favorable electrostatic interactions with cell membrane fragments and facilitates the proper orientation of the membrane during the coating procedure. This orientation is crucial for preserving the biological functionality of the membrane proteins. The zeta potential of the resulting MFS NSs was determined to be −12.3 ± 0.21 mV. Such a slightly negative, near‐neutral surface is favorable for in vivo applications, as it reduces non‐specific protein adsorption and macrophage recognition, thereby supporting prolonged blood circulation.

**FIGURE 4:**
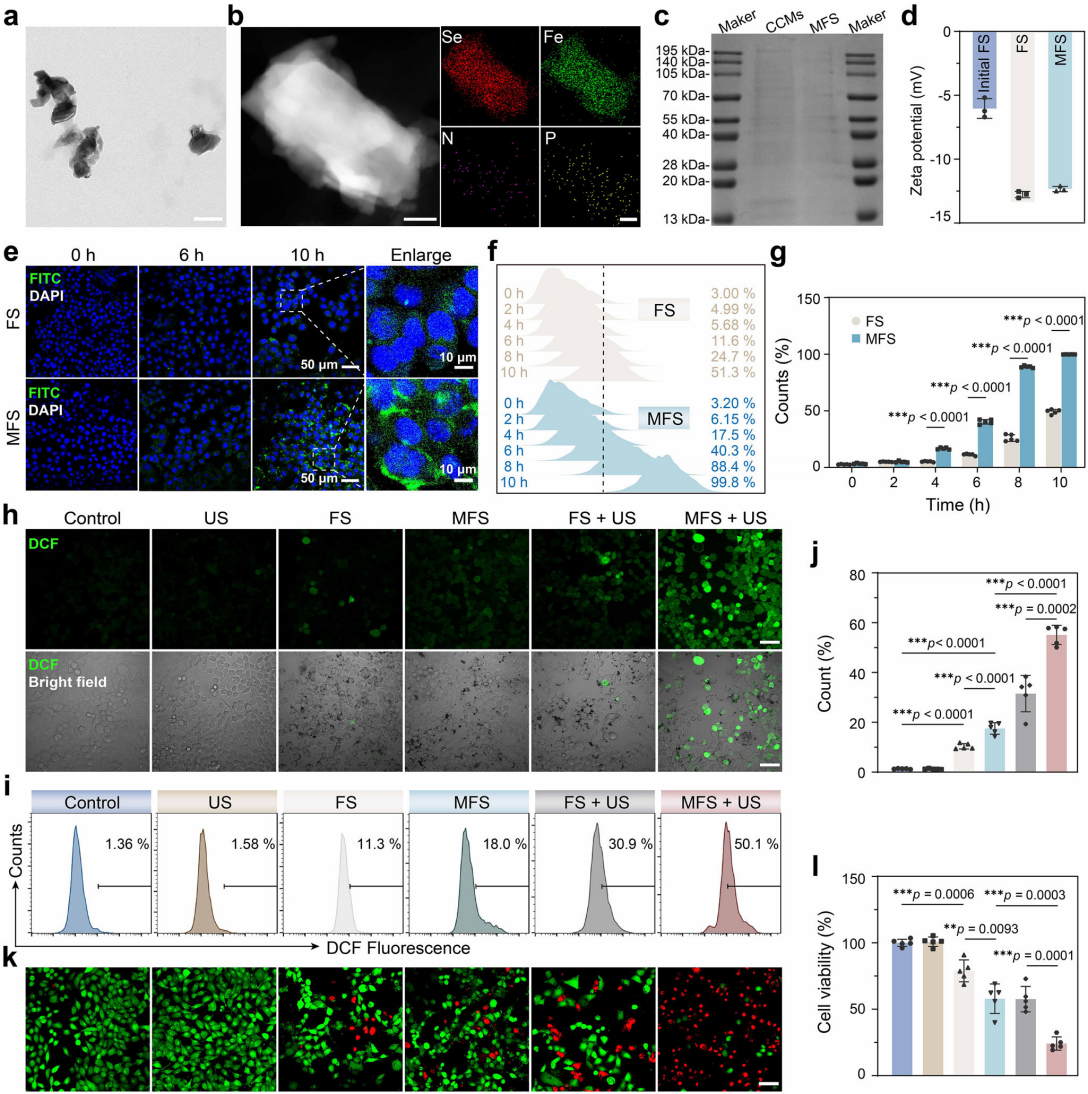
Evaluation of cellular internalization, oxidative stress induction, and cytotoxic potential of MFS in vitro. (a) The TEM image of MFS NSs (scale bar: 200 nm). (b) The elemental mapping images of MFS NSs (scale bar: 50 nm). (c) SDS‐PAGE protein analysis of 4T1 CCMs and MFS NSs. (d) Zeta potential of initial FS NSs, FS NSs (Initial FS NSs modified with mPEG‐NH_2_), and MFS NSs. (e) Representative CLSM images of 4T1 cells after co‐incubated with FITC‐labeled FS or MFS NSs after 0, 6, and 10 h. (f) FCM assay of cellular uptake performance and (g) the corresponding statistical analysis at different time points (*n* = 5). (h) Representative CLSM images showing the ROS generation in 4T1 cells at various treatments (scale bar: 50 µm). (i) Representative FCM analysis plots illustrating the intracellular ROS generation and (j) the corresponding fluorescence intensity at various treatments (*n* = 5). (k) CLSM images of 4T1 cells stained with Calcein‐AM (green, indicating the living cells) and PI (red, indicating the dead cells), after different treatments (scale bar: 50 µm). (l) CCK‐8 assay quantified the viability of 4T1 cells after different treatments (*n* = 5).

Prior to the evaluation of the performance and biomedical applications, the biocompatibility of MFS NSs was systematically investigated. To this end, a series of hematological analyses were conducted using fresh whole blood samples. Compared with the control group, the standard hematological parameters and biochemical markers in the murine blood of the drug injection group remained within the confines of the normal physiological spectrum (Figure S6a). Furthermore, histopathological examination of tissue sections, stained with Hematoxylin and Eosin (H&E), indicated that the cellular architecture of the major organs maintained their typical morphology, exhibiting no significant pathological modifications (Figure S6b). The comprehensive hematological assessments and histopathological examination results collectively substantiate the biocompatibility and non‐toxicological profile of MFS NSs in vivo. Consequently, these observations provide a robust foundational premise for the prospective utilization of MFS NSs in further biomedical applications, prompting subsequent investigative endeavors to delineate their potential in therapeutic interventions.

### 2.6 Evaluation of Cellular Internalization, Oxidative Stress Induction, and Cytotoxic Potential of MFS In Vitro

The homologous targeting capability of the membrane‐coated nanosheets was further evaluated. The cellular uptake of FITC‐labeled FS and MFS NSs by 4T1 cells was visually tracked through confocal laser scanning microscopy (CLSM) imaging and flow cytometry (FCM) analysis. As shown in Figure 4e, the green fluorescence signal increased over time, and a more significant fluorescence signal appeared around the 4T1 cell nucleus in the MFS group was observed, indicating the specific binding ability of MFS NSs towards homologous 4T1 cancer cells. Furthermore, quantitative FCM analysis revealed that the uptake efficiency of MFS NSs by 4T1 cells approached nearly 100% after 10 h, approximately twice that of the FS group (Figure 4f,g).

Given the high internalization capacity of MFS NSs by 4T1 cells, we conducted an in‐depth exploration of their therapeutic potential in subsequent intracellular applications. The ultrasonic catalytic properties of MFS NSs, in conjunction with the Fenton‐like reaction of iron ions, synergistically induce excessive intracellular ROS generation, ultimately resulting in mitochondrial dysfunction and potent cytotoxicity [46]. Intracellular ROS levels were observed using the DCFH‐DA fluorescent probe through CLSM and FCM analysis. The CLSM images revealed minimal fluorescence in both the control and US groups, whereas trace fluorescence was detected in the FS and MFS groups. Notably, moderate fluorescence emerged in the FS group upon ultrasonic stimulation. In stark contrast, the strongest fluorescence was observed in 4T1 cells treated with MFS under US stimulation (Figure 4h). These findings suggest that the combined treatment of MFS + US significantly elevated the intracellular level of ROS during SDT and CDT, a conclusion further substantiated by quantitative FCM analysis (Figure 4i,j).

Subsequently, to gain more profound insights into corroborating the cytotoxicity results, live/dead cell dual staining tests were performed using calcein acetoxymethyl ester (Calcein‐AM) and propidium iodide (PI) probes. As illustrated in Figure 4k, a notable increase in red fluorescence, indicative of dead cells, was observed following treatment with MFS NSs under US conditions. Conversely, a significant enhancement in green fluorescence, representing viable cells, was evident in the other groups. These findings were consistent with the results obtained from = 88 assays (Figure 4l). Collectively, the aforementioned cytotoxicity assessments demonstrated that the combination of MFS NSs with US serves as a highly efficacious platform for tumor therapy.

### 2.7 In Vitro Assessment of MFS‐Induced Ferroptosis

The aforementioned experiments have conclusively demonstrated that the substantial quantity of ROS generated through the synergistic effect of SDT and CDT exhibited marked cytotoxicity. However, the overexpressed GSH in cancer cells helps to maintain the redox homeostasis necessary for proliferation by consuming ROS, and promotes the expression of glutathione peroxidase 4 (GPX4) [47]. The activation of GPX4 by GSH further enhances the antioxidant defense mechanisms in tumor cells, making them more resistant to antitumor therapies. Therefore, the depletion of GSH within tumor cells carries considerable importance. MFS NSs exhibited a responsive release of Fe^2+^ within acidic microenvironment of tumor cells, leading to the oxidation of Fe^2+^ to Fe^3+^ through the Fenton reaction, accompanied by the production of ROS. The Fe^3+^ generated can be reductively converted back to Fe^2+^ by GSH, which can then participate in the Fenton reaction again, while consuming GSH and achieving ferroptosis of cancer cells (Figure 5a). Firstly, we quantitatively analyzed the Fe^2+^ content within 4T1 cancer cells using a Ferrous Ion Content Detection Kit. The findings indicate an elevation in intracellular Fe^2+^ content following FS NSs treatment, whereas a marked accumulation of Fe^2+^ was observed in cells subjected to MFS NSs treatment (Figure 5b). Accordingly, a decline in intracellular GSH levels was observed following treatment with FS NSs, with a more pronounced reduction noted after MFS NSs treatment. Notably, the greatest decrement in GSH content was achieved when combined with US irradiation (Figure 5c). The intracellular activity of GPX4 exhibited a comparable trend, with the lowest activity observed in the group subjected to MFS NSs in conjunction with US irradiation (Figure 5d). Additionally, the expression level of GPX4 in 4T1 cancer cells was further examined through western blot analysis. When compared to the control group, both FS NSs and MFS NSs treatment groups exhibited a moderate decrease in GPX4 expression. Notably, the MFS NSs + US group demonstrated the most significant downregulation of GPX4 expression (Figure 5e,f). Given the heightened ROS production, GSH depletion, and GPX4 inactivation capabilities, the lipid peroxidation (LPO) levels within 4T1 cells were subject to further assessment [48]. C11‐BODIPY^581/591^, a fluorescent probe exhibiting high sensitivity and specificity for detecting LPO, undergoes a shift from red to green upon selective capture of lipid peroxides. The CLSM images provided visual evidence of moderate green fluorescence following treatment with FS NSs and MFS NSs, whereas the MFS NSs + US group demonstrates a pronounced bright green fluorescence (Figure 5g). In addition, we tested the intracellular lipid peroxide malondialdehyde (MDA) levels of 4T1 cells that were treated with various groups, and the results showed that MFS NSs + US group significantly enhanced MDA levels in 4T1 cells (Figure 5h).

**FIGURE 5:**
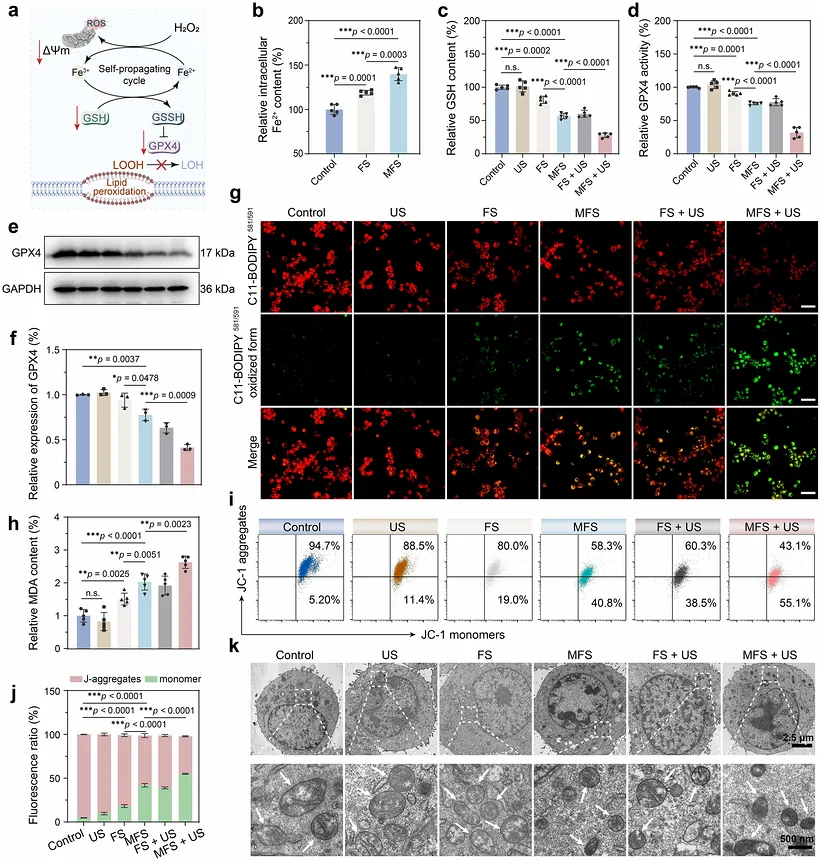
Intracellular ferroptosis inducement. (a) Schematic illustration of the underlying mechanisms and consequent effects of ferroptosis induction in cancer cells. (b) Fe^2+^ content in 4T1 cells after various treatments (*n* = 5). (c) Relative GSH levels in 4T1 cells after different treatments (*n* = 5). (d) Relative GPX4 activity in 4T1 cells after different treatments (*n* = 5). (e) Western blot analysis of GPX4 protein expression with varied treatment conditions and (f) the corresponding quantitative analysis (*n* = 3). (g) Representative CLSM images of C11‐BODIPY^581/591^ dye‐stained 4T1 cells after various treatments (scale bar: 50 µm). (h) The intracellular LPO product MDA detection after different treatment conditions (*n* = 5). (i) Representative FCM analysis plots of the mitochondria membrane potential following different treatments and (j) the quantitative analysis of JC‐1 aggregates and monomers in 4T1 cells (*n* = 5). (k) TEM images of 4T1 cells following various treatments, with white arrows pointing to the mitochondria.

Subsequently, we conducted an in‐depth investigation of mitochondrial alterations in response to elevated intracellular oxidative stress. First, the mitochondrial membrane potential was assayed using JC‐1 dye. The CLSM images revealed a notable decrease in the intensity of JC‐1 aggregates (exhibiting red fluorescence) following treatment, while a marked increase was observed in the intensity of JC‐1 monomers (emitting green fluorescence) (Figure S7). These findings suggest a pronounced decline in mitochondrial membrane potential. Notably, the MFS + US group exhibited the most substantial reduction in mitochondrial membrane potential. Meanwhile, the quantitative assessment of mitochondrial membrane potential through FCM analysis demonstrated a consistent trend (Figure 5i,j). Additionally, TEM was employed to observe the morphological alterations in the mitochondria of 4T1 cells across various treatment groups. Compared to the control and US group, the mitochondria in 4T1 cells from multiple treatment groups demonstrated varying levels of morphological abnormalities (Figure 5k). Notably, the mitochondria in the 4T1 cells subjected to the MFS + US regimen exhibited a substantial shrinkage in volume, coupled with an elevation in the density of the bilayer membrane and a reduction in the number of mitochondrial cristae, which are consistent with the characteristics of mitochondrial dysfunction induced by ferroptosis. To definitively confirm the occurrence of ferroptosis, several typical ferroptosis inhibitors were incorporated into the treatment regimen. Ethylenediaminetetraacetic acid, functioning as a chelator, diminishes iron‐catalyzed lipid peroxidation by sequestering free iron ions within 4T1 cells. And ferrostatin‐1 disrupts the ferroptosis pathway by inhibiting lipid peroxidation chain reactions [49]. Meanwhile, GSH and vitamin E, crucial intracellular antioxidants, neutralize lipid peroxides, alleviate oxidative stress, and consequently inhibit ferroptosis indirectly [47, 50]. The Cell Counting Kit‐8 (CCK‐8) assay results demonstrated a significant reduction in cytotoxicity induced by the MFS + US treatment after inhibiting selected critical stages of the ferroptosis process, thereby confirming the induction of ferroptosis (Figure S8). Additionally, a selection of inhibitors specific to canonical cell death pathways were introduced into the system for the purpose of distinguishing apoptosis, autophagy, and necrosis, thereby validating the therapeutic contribution of ferroptosis (Figure S9). The application of the apoptosis inhibitor Z‐VAD‐FMK demonstrated a partial attenuation of cytotoxicity, suggesting the existence of an apoptotic‐inducing capacity within the generated ROS. Conversely, neither the autophagy inhibitor 3‐methyladenine nor the necrosis inhibitor necrostatin‐1 exhibited any appreciable influence on cellular viability.

### 2.8 Antibacterial Activity of MFS Against Intracellular Bacteria in Tumor Cells

TRIB exert profound influences on the proliferation, metabolism, and invasive capabilities of cancer cells, and serve as pivotal factors in facilitating tumor progression and enhancing metastasis. This revelation challenges conventional paradigms in cancer therapeutic strategies, underscoring the significance of focus on nontumor cellular components within the tumor microenvironment. Therefore, we simulated the condition of cancer cells harboring bacteria within tumor tissues by inoculating *S. aureus* into 4T1 cellular models. Given that direct physical contact between nanomaterials and bacteria is a prerequisite for effective intracellular antibacterial therapy, we first verified whether MFS NSs could reach and contact the intracellular *S. aureus*. To track the intracellular fate of MFS NSs, the co‐localization of FITC‐labeled MFS with Lyso‐Tracker‐labeled lysosomes was examined at 0.5, 3, and 12 h (Figure S10a). MFS was initially associated with the cell membrane (0.5 h), showed pronounced lysosomal co‐localization (3 h), and subsequently became partially dispersed in the cytoplasm (12 h), suggesting that endocytosis, lysosomal trafficking, and cytosolic release may proceed concurrently. The persistence of partial lysosomal co‐localization alongside the growing cytosolic signal implies that MFS NSs may gradually escape from lysosomes rather than through a single, one‐step release event, potentially via lysosomal membrane disruption or the proton‐sponge effect. Meanwhile, the possibility that a fraction of nanosheets enters the cytoplasm through non‐endocytic routes, such as direct membrane translocation, cannot be excluded. Further studies are warranted to elucidate the detailed mechanism underlying the cytosolic delivery of MFS NSs. 4T1 cells harboring FITC‐labeled *S. aureus* were incubated with DiI‐labeled MFS NSs and subsequently examined by CLSM. As shown in Figure S10b, the red fluorescence of DiI‐labeled MFS NSs displayed punctate distribution in the cytoplasm, and part of the green fluorescent *S. aureus* was found to be surrounded by, or in close proximity to, the red MFS signals. This spatial overlap indicates that MFS NSs can directly contact the intracellular bacteria after being internalized by tumor cells, laying the foundation for their intracellular antibacterial action.

Subsequently, we conducted a series of experiments to assess the bactericidal capacity of MFS NSs against intracellular bacteria (Figure 6a). The representative digital photos obtained through the plate spreading method of bacteria harvested from 4T1 cells infected with *S. aureus* revealed a marked decrease in colony counts within the MFS + US treatment group when compared to other control groups, thereby exhibiting a pronounced bactericidal effect (Figure 6b). The quantitative viability results, calculated based on colony counts, demonstrated a substantial enhancement in bactericidal efficacy for the MFS + US group when compared to the MFS group, thereby suggesting a notable contribution from SDT (Figure 6c). Furthermore, the FCM analysis was further employed to evaluate the content of FITC‐labeled *S. aureus* within 4T1 cells (Figure 6d,e). After treatment with FS NSs and MFS NSs, there was a modest reduction in the intracellular bacterial load within the 4T1 cells, corresponding to 54.2% and 45.8%, respectively. Following combination with US irradiation, a marked decrease in bacterial content was observed in the FS + US treatment group, plummeting to 16.8%. Notably, the MFS + US treatment group almost achieved the complete eradication of intracellular bacteria, with the bacterial load decreasing to a mere 4.89%, further illustrating the importance of enhanced cellular uptake in potentiating the therapeutic efficacy. Additionally, the CLSM images provided a visual representation of a substantial reduction in green fluorescence, indicative of the internalized *S. aureus*, within the MFS group and FS + US group, whereas the MFS + US treatment group exhibited negligible fluorescence (Figure 6f). Further TEM analysis reinforced that a marked decrease in the intracellular count of *S. aureus* in the MFS + US treatment group, accompanied by structural damage, indicating robust inhibitory effects (Figure 6g). The findings demonstrate that the substantial production of ROS, mediated by the US‐triggered catalytic phenomenon associated with MFS NSs, results in significant cytotoxic and bactericidal effects.

**FIGURE 6:**
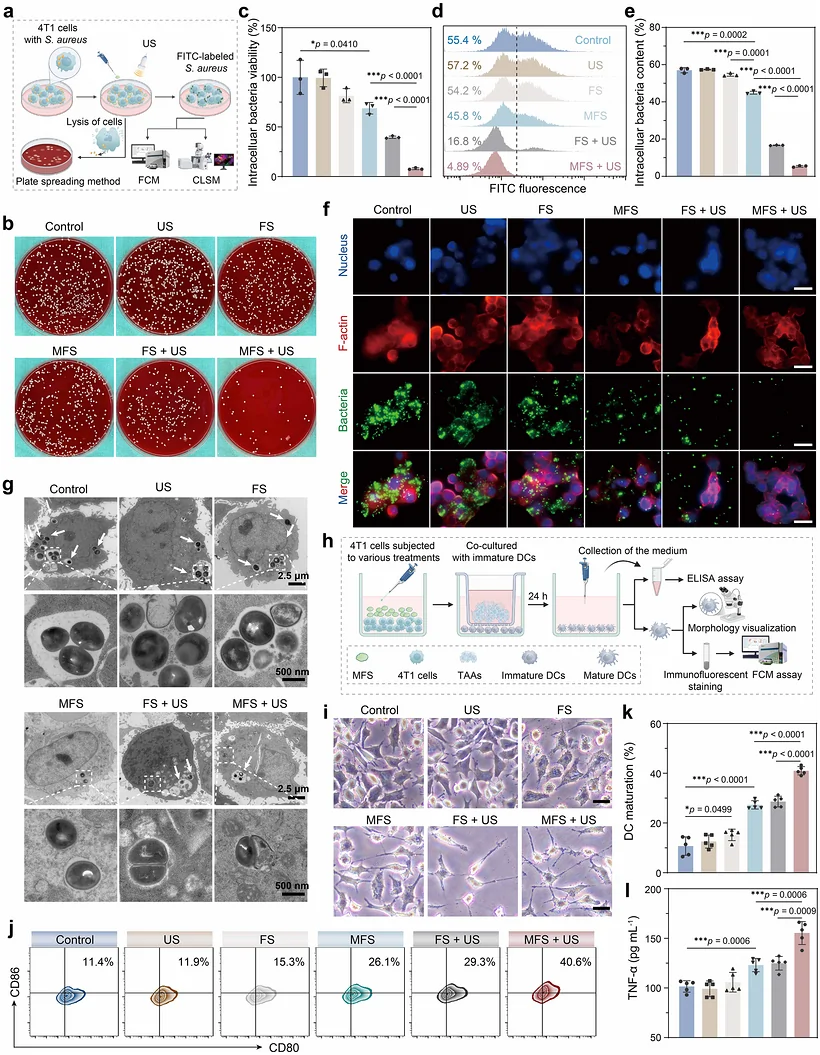
Antibacterial activity and induction of DC maturation in vitro. (a) Schematic diagram showing the protocol for assessing the bactericidal efficacy of MFS NSs against intracellular bacteria. (b) Representative photos of blood agar plate spreads after various treatments and (c) the corresponding viability of *S. aureus* determined by colony counting (*n* = 3). (d) Representative FCM analysis plots illustrating the intracellular *S. aureus* content after different treatments (e) and the corresponding quantitative analysis (*n* = 3). (f) Representative CLSM images of intracellular *S. aureus* staining following various treatments (scale bar: 25 µm). (g) Representative TEM images of intracellular *S. aureus* following different treatment conditions, with white arrows indicating the bacteria. (h) A schematic illustration outlining the protocol for inducing the maturation of DCs in vitro. (i) Representative cell morphology images of DCs co‐incubated with 4T1 cells subjected to various treatments (scale bar: 50 µm). (j) Representative FCM analysis plots and (k) relative quantification of DC maturation (gated at CD11c^+^ CD80^+^ CD86^+^). (l) The expression levels of TNF‐𝛼 secreted from DC2.4 cells co‐incubating with 4T1 cells after various treatments (*n* = 5).

### 2.9 In Vitro Activation of the Immune Response

Cells experiencing ferroptosis are known to stimulate an anti‐tumor immune response by releasing DAMPs, which encompass the translocation of calreticulin (CRT) and the secretion of high mobility group box 1 (HMGB‐1) [29]. These processes collectively contribute to the maturation of dendritic cells (DCs). As depicted in Figure S11a, both the MFS and FS + US groups elicited a moderate expression of CRT on the cellular membrane. Notably, the combinatorial treatment regimen, MFS + US, demonstrated the most profound effect on CRT exposure among all treated groups. Similarly, regarding the release of HMGB‐1 from the nucleus, the MFS + US group also exhibited the most marked increase compared to all other experimental conditions (Figure S11b). The released DAMPs can facilitate the enhancement of the maturation process of immature DCs. Matured DCs exhibit upregulated expression of co‐stimulatory molecules, such as CD80 and CD86, and secrete cytokines, including TNF‐α and IL‐6, which collectively enhance T‐cell activation and facilitate the initiation of adaptive immune responses (Figure 6h). Therefore, it was reasonable to hypothesize that the release of DAMPs, induced by the inhibitory effect of MFS NSs against cancer cells, possessed the potential to induce DC maturation. First, the optical microscope photographs provided a visual representation of the morphological changes in DCs during their maturation process. DCs in the MFS and FS + US groups progressively displayed irregular shapes, with the emergence of dendrite‐like protrusions on their surfaces. Notably, DCs from the MFS + US group demonstrate the most advanced features indicative of differentiation and maturation (Figure 6i). Subsequently, we further examined the DC maturation markers to evaluate the DC maturation levels through FCM analysis. The expression levels of CD80 and CD86 in the MFS + US pretreatment group were significantly increased, being much higher than those in the other groups (Figure 6j,k). Additionally, the MFS + US group exhibited the highest concentrations of TNF‐α and IL‐6 cytokines (Figure 6l and Figure S12). Collectively, the pretreatment of 4T1 cells with MFS NSs‐based intervention stimulates the activation of DCs, thereby enhancing the presentation of tumor antigens.

To further investigate the contribution of bacteria‐derived PAMPs in the complete system, *S. aureus* was introduced into the co‐culture system, and the DC2.4 maturation levels were assessed through FCM analysis (Figure S13). The results demonstrated that DCs co‐incubated with *S. aureus* + MFS + US group exhibited moderate maturation (23.6% on average), being higher than that in the untreated control group (16.1%), which verified that PAMPs alone possessed the capacity to activate DCs. In comparison, DCs co‐incubated with 4T1 + MFS + US group displayed a stronger response (33.7%), further confirming that DAMPs served as the dominant activation signal. Notably, when *S. aureus*‐harboring 4T1 cells were treated with antibiotics to remove the TRIB, the DC maturation was significantly reduced (40.6%), being much lower than that in the *S. aureus*‐harboring 4T1 + MFS + US group without antibiotic treatment (54.6%) and close to the DAMPs‐only level. Collectively, these results demonstrate that the introduction of bacteria provides a significant additional contribution to DC maturation beyond tumor cell death‐derived DAMPs, thereby further enhancing the presentation of tumor antigens.

### 2.10 Biodistribution of MFS NSs

Achieving a high concentration of therapeutic agents within tumor tissues constitutes a fundamental prerequisite for enhancing treatment efficacy. Furthermore, the targeting capability of these agents towards tumor tissues is pivotal in mitigating unintended damage to normal tissues. Within the framework of SDT, the imaging capability of therapeutic agents within tumor tissues holds paramount significance. Imaging technologies facilitate visual monitoring, enabling the tracking of agent infiltration into tumor regions and the temporal profile of their accumulation, thereby furnishing robust support for the precise spatial and temporal control of US irradiation therapy. This approach ensures the precise application of external energy therapy within designated target regions confined to specific temporal and spatial boundaries, ultimately leading to enhanced optimization and substantial augmentation of the overall therapeutic outcome.

Given the negligible systemic toxicity of MFS NSs, we conducted an in‐depth assessment of their imaging capabilities within tumor locations. This enabled the visualization and monitoring of MFS NSs accumulation within tumor tissues, ultimately serving as a foundational guide for subsequent therapeutic interventions. Firstly, we evaluated the photoacoustic (PA) imaging capability of MFS NSs in vitro. Through the acquisition of PA images spanning the wavelength range of 680 nm–790 nm, our results demonstrated that MFS NSs possess remarkable PA imaging capabilities, with the peak PA intensity observed at an excitation wavelength of 715 nm (Figure S14). The intensity of the PA signal exhibited a pronounced dose‐dependent relationship with MFS NSs, demonstrating an excellent linear correlation (Figure S15).

Subsequently, we further evaluated the PA imaging capability of nanosheets within tumor regions. The PA signal intensity within the tumor region demonstrated a progressive enhancement following intravenous administration, correlating with the duration post‐injection. Notably, the PA signal intensity in tumors treated with MFS NSs was significantly higher compared to those treated with FS NSs, with the difference becoming more pronounced as time progressed (Figure S16a). Furthermore, the quantitative analysis indicated that at 8 h postintravenous injection, the PA signal intensity in the tumor tissue of the FS NSs group peaked at 0.377 ± 0.097. In comparison, the MFS NSs group exhibited a significantly higher peak intensity at 0.582 ± 0.026, indicative of a more pronounced accumulation of the modified nanosheets at the tumor site (Figure S16b). These findings suggested that the surface modification of the FS NSs with CCMs constituents significantly enhances the targeting efficiency of the nanosheets, thereby promoting their preferential accumulation within the tumor microenvironment. Additionally, the excellent PA imaging performance of these nanosheets in the tumor regions can serve as a crucial indicator for guiding ultrasonic irradiation therapy to achieve precise treatment.

Given the PA imaging results that demonstrate the capability of MFS NSs to accumulate predominantly within tumor tissues, the fluorescence‐based imaging technique was further employed to delineate in vivo biodistribution, thereby offering further validation of the targeting performance modified by tumor cell membranes. The fluorescence signal of the Cy5.5‐labeled nanosheets in the tumor region exhibited a trend consistent with the PA signal, with a progressive increase of the fluorescent signal at the tumor region as a function of post‐injection time, culminating in a maximum intensity at 8 h (Figure S17a). Furthermore, the administration group receiving Cy5.5‐MFS NSs exhibited a markedly intensified fluorescence signal in the tumor region when contrasted with the Cy5.5‐FS NSs, further corroborating their enhanced accumulation efficacy in the tumor tissue. However, following the injection of Cy5.5‐FS NSs into tumor‐bearing mice, it was observed that they underwent more rapid excretion via the urinary system and were subsequently eliminated from the body. Concurrently, 8 h postinjection, the tumor tissues were harvested for frozen section analysis (Figure S17b). There was a notably higher fluorescence signal intensity within the tumor tissues for DiR‐MFS NSs compared to DiR‐FS NSs.

### 2.11 Antitumor Efficiency In Vivo

Encouraged by the promising results of immune stimulation in vitro, the therapeutic application of MFS NSs for antitumor therapy was further evaluated under the guidance of the imaging results (Figure 7a). 4T1 tumor‐bearing mice were randomly divided into 6 groups and subjected to the following treatments: (1) Control; (2) US; (3) FS; (4) MFS; (5) FS + US; (6) MFS + US. During the treatment period, precise measurements and documentation of the body weight and tumor volumes were conducted every other day. Tumor growth volume monitoring data indicated a rapid increase in tumor size within the monitoring period for both the Control and US irradiation groups, with average volumes reaching 670.60 mm^3^ and 619.99 mm^3^ on day 20, respectively (Figure 7b,c). The administration of FS NSs exhibited a modest inhibitory impact on the increase of tumor volume (average volume: 486.97 mm^3^). However, a notable enhancement in inhibitory efficacy was observed in the groups treated with MFS NSs and FS in combination with US therapy, reaching average volumes of 246.79 mm^3^ and 253.41 mm^3^ respectively. These results demonstrate that surface modification significantly enhances tumor‐targeting efficacy, and the SDT induced by US irradiation further enhances the therapeutic antitumor response. Encouragingly, the MFS + US group showed a modest reduction in tumor volume, with average volumes reduced to 63.97 mm^3^, signifying an enhanced efficacy in inhibiting tumor proliferation. Compared to the MFS + US group, the FS + US group demonstrated a marked decrement in antitumor efficacy, attributable to insufficient tumor accumulation. Concurrently, the MFS group, lacking the augmentation of sonodynamic effects, also exhibited inferior therapeutic performance. The average tumor weight in the MFS + US treatment group on the 20th day was significantly reduced, exhibiting a decrement of approximately 13.34‐, 10.78‐, 5.53‐, 3.73‐, and 4.13‐fold when compared to the tumor weights in the Control, US, FS, MFS, and FS + US groups, respectively (Figure 7d,e).

**FIGURE 7:**
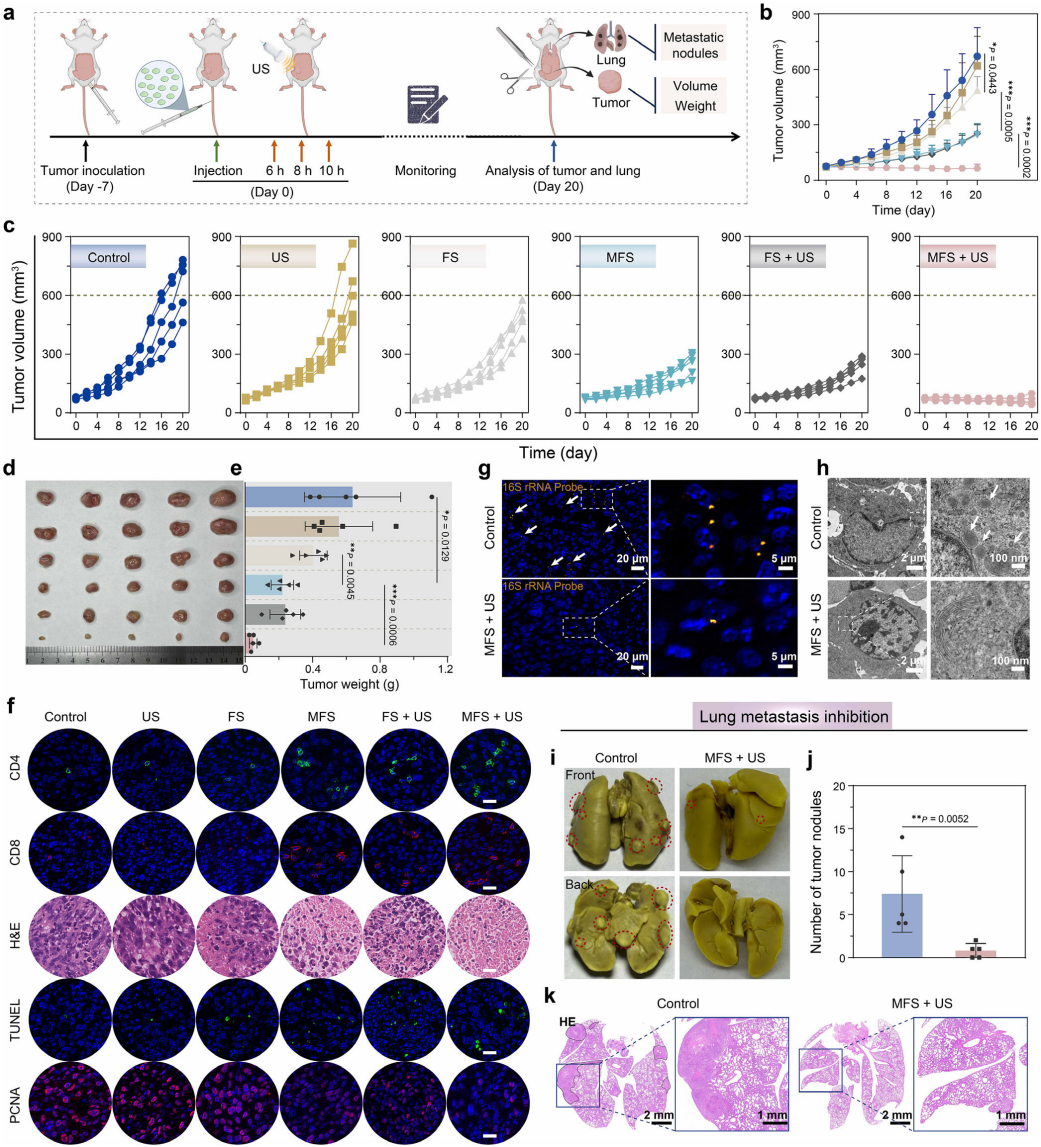
Evaluation of antitumor and antibacterial efficiency, and assessment of metastasis inhibition. (a) Schematic representation of the MFS‐based therapeutic approach to suppress tumor proliferation and metastasis. (b) The average tumor volume curve after different treatments (*n* = 5). (c) The individual tumor volume curve of each group after different treatments (*n* = 5). (d) Weight and (e) digital photos of excised tumor tissues from the 4T1 tumor‐bearing mice subsequent to various treatment (*n* = 5). (f) CD4, CD8, H&E, TUNEL, and PCNA staining of tumor tissues (scale bar: 20 µm). (g) Immunofluorescence staining with 16S rRNA FISH probe on tumor sections, with a white arrow indicating the presence of 16S positive bacteria. (h) Representative TEM images depicting the bacterial structures localized within the cytosol of tumor cells. (i) Representative digital photos of excised lung tissue in the control group and MFS + US group. (j) Number of visible tumor nodules in lung tissues between control and MFS + US groups (*n* = 5). (k) Representative images of H&E‐stained lung tissues.

To further investigate the mechanisms of therapy, a random sample from the excised tumor tissues of each group was subjected to immunofluorescence analysis with CD4 and CD8 antibodies, as well as to histopathological evaluation through H&E staining. Additionally, apoptosis was assessed by terminal‐deoxynucleotidyl transferase‐mediated dUTP Nick‐End labeling (TUNEL) staining, and cellular proliferation was examined using proliferating cell nuclear antigen (PCNA) staining (Figure 7f). The results revealed the highest fluorescence intensity associated with positive CD4 and CD8 antibody staining in the MFS + US group, signifying a significant infiltration of T cells with antitumor activity. Similarly, the group exhibited the highest green fluorescence with positive TUNEL staining, indicative of a pronounced extent of apoptosis and necrosis in tumor cells. Conversely, the lowest red fluorescence with positive PCNA staining was observed, suggesting a reduced level of tumor cell proliferation. These findings are in concordance with the previously documented enhanced antitumor efficacy of the MFS + US therapeutic regimen. No reduction in body weight was observed throughout the duration of the therapeutic period (Figure S18), further indicating the favorable safety profile of the therapeutic approach.

### 2.12 Eliminating TRIB Enhanced the Inhibition of Tumor Growth and Metastasis

Researches have demonstrated that the targeted depletion of TRIB can markedly suppress the distant metastasis of tumors. Considering the pronounced bactericidal efficacy demonstrated by the MFS nanosheet‐based therapeutic regimen in vitro, further investigation into the strategy for eliminating TRIB to achieve the suppression of tumor metastasis has been conducted (Figure 7a). On the 20th day following the aforementioned treatments, a random sample from the collected tumor tissues of the Control group and the MFS + US group was subjected to immunofluorescence analysis combined with 16S fluorescence in situ hybridization (FISH). Within the tumor sections of the Control group, a significant presence of 16S rRNA‐positive fluorescence was detected, which is indicative of the presence of highly conserved bacterial genes (Figure 7g). However, there was almost no fluorescence in the MFS + US treatment group. Subsequently, the bacterial co‐localization within the tumor tissue was further investigated via Bio‐TEM technique. Representative TEM micrographs demonstrated that a significant majority of bacterium‐like structures were predominantly identified within the cytoplasmic compartments of the tumor cells, and the observed quantity corresponded with the results of the 16S rRNA FISH analysis (Figure 7h). These results demonstrate that the therapeutic strategy employed exhibits a substantial lethal effect on TRIB, highlighting its potential efficacy in the targeted eliminating of this specific biological entity.

Following the aforementioned therapeutic intervention, we examined the influence of TRIB on tumor metastasis by evaluating the incidence of pulmonary metastases in the treated mice. Subsequent to the euthanasia of the mice, pulmonary specimens were collected and fixed with Bouin's fixative solution, facilitating the identification of metastatic nodules within the lung tissue. As evidenced by the representative photographs and quantitative analysis of the excised pulmonary specimens, the MFS + US treatment group exhibited a negligible incidence of pulmonary metastasis (Figure 7i). Rather remarkably, a severe burden of pulmonary metastasis was observed in the Control group, characterized by a substantial elevation in the number of nodules, which was 8.5‐fold higher compared to the treatment group (Figure 7j). Additionally, the corresponding H&E staining provided a clear visualization of the metastatic nodules in the lung. These histological sections illustrated the extensive tumor pathologies within the lung parenchyma of the Control group, in marked contrast to the negligible incidence observed in the MFS + US treatment group (Figure 7k). The findings were in concordance with the anticipated results, demonstrating that the intervention strategy markedly decreased the probability of lung metastasis originating from the orthotopic tumor. The potential mechanisms responsible for the therapeutic efficacy observed can be ascribed to two distinct reasons. Firstly, the intervention is hypothesized to reduce the risk of lung metastasis by eliminating TRIB, a hypothesis that is supported by the scientific literature and experimental observations. Subsequently, a secondary proposed mechanism involves the induction of systemic anti‐tumor immune responses. In vitro cell experiments provide convincing evidence to suggest that the intervention strategy has the capacity to significantly stimulate the immune response.

### 2.13 Systemic Antitumor Immune Activation

To investigate the underlying immunological mechanisms responsible for the pronounced inhibition of tumor growth and metastasis observed during therapy, we subsequently embarked on an exhaustive in vivo immunological profiling (Figure 8a). Encouraged by the results from in vitro cellular assays, the application of MFS + US strategy was demonstrated to potently induce ICD and intracellular bacterial death within 4T1 cells. This process facilitated the secretion of DAMPs and PAMPs, subsequently inducing the maturation of DCs. Therefore, the proportion of mature DCs in tumor tissue, sentinel lymph nodes (SLN), and splenic tissues was evaluated through FCM analysis. Compared to untreated mice and mice treated with US, FS, MFS or FS + US, mice treated with MFS + US strategy exhibited the highest proportion of mature DCs (gated on CD11c^+^CD80^+^CD86^+^) in both tumor tissues, SLN, and splenic tissues, which were 59.62 ± 3.82%, 67.06 ± 0.86%, and 48.30 ± 0.60%, respectively (Figure 8b–e). Medium levels of DC maturation were detected in MFS group (49.14 ± 4.09%, 58.70 ± 1.45%, 42.36 ± 4.03%, respectively) and FS + US group (50.04 ± 5.56%, 56.90 ± 6.51%, 40.96 ± 1.99%, respectively. Simultaneously, the MFS + US treatment group also exhibited the highest levels of secretion for the characteristic pro‐inflammatory cytokines TNF‐α and IL‐6 in the serum (Figure 8f,g). The observed results were consistent with the predictions emanating from the in vitro studies on immune activation and antibacterial efficacy.

**FIGURE 8:**
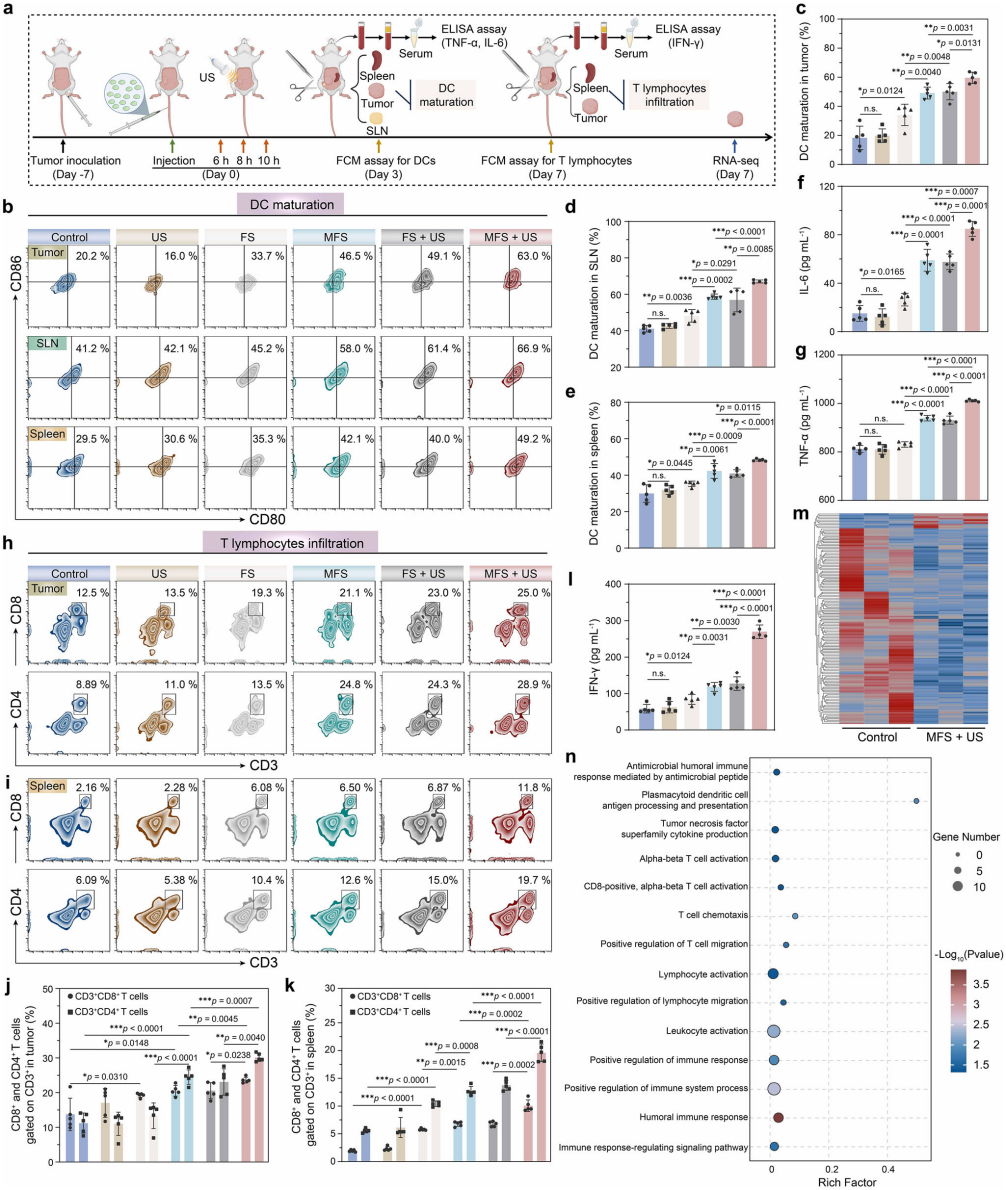
Systemic immune sequential activation and underlying mechanisms. (a) Schematic illustration of the protocol for assessing the activation of systemic antitumor immune responses and investigating the potential mechanism. (b) Representative flow cytometric results and (c–e) corresponding quantitative analysis of mature DCs (gated on CD11c^+^CD80^+^CD86^+^) in tumor, SLN, and spleen of 4T1 tumor‐bearing mice after various treatments (*n* = 5). (f, g) Quantification of serum cytokine levels for IL‐6 and TNF‐α following different therapeutic interventions (*n* = 5). (h, i) Representative flow cytometric results and (j, k) corresponding quantitative analysis of T lymphocytes (helper T lymphocytes gated on CD3^+^CD4^+^, CTLs gated on CD3^+^CD8^+^) infiltration in tumor and spleen of 4T1 tumor‐bearing mice after various treatments (*n* = 5). (l) Quantification of serum cytokine levels for IFN‐γ after various treatments (*n* = 5). (m) Heatmap of DEGs between Control group and MFS + US group (*n* = 3). (n) GO enrichment bubble diagrams highlighting the expression of biological pathways linked to antitumor immune defense mechanisms.

Mature DCs, serving as a pivotal component in the immune response, can effectively present tumor‐associated antigens via major histocompatibility complex molecules to T lymphocytes. This presentation stimulates the proliferation and activation of T lymphocytes, ultimately triggering anti‐tumor immune‐killing effects. Following the prior analysis, the infiltration of T lymphocytes within tumor and splenic tissues was also evaluated through FCM. CD8^+^ cytotoxic T lymphocytes (CTLs) and CD4^+^ helper T lymphocytes are recognized as potent effector cells in the antitumor immune response, playing critical roles in the recognition and elimination of tumor cells. Encouragingly, a maximum infiltration of both CTLs and CD4^+^ helper T lymphocytes was observed in the tumor tissues of the MFS + US treatment group, with infiltration rates of 23.62 ± 0.83% and 30.16 ± 1.18%, respectively (Figure 8h,j). Notably, as a pivotal organ in systemic immune regulation, spleen exhibited a marked enhancement in the infiltration of CTLs and CD4^+^ helper T lymphocytes post‐therapy. The proportions of these lymphocytes in the spleen tissue were elevated to 5.19‐fold and 3.53‐fold compared to the Control group, respectively (Figure 8i,k). Concurrently, there was a marked elevation in the level of IFN‐γ within the serum, a pro‐inflammatory cytokine predominantly secreted by helper T lymphocytes and CTLs (Figure 8l). The results suggest that the combinatory treatment elicits a robust in situ anti‐tumor immune response while also amplifying a significant systemic immune reaction. Furthermore, these findings offer compelling immunological evidence to support the observed inhibition of tumor growth and metastasis in the aforementioned study.

To further assess whether the MFS + US treatment could confer long‐term immune protection against tumor metastasis, the memory phenotypes of splenic T lymphocytes were subsequently interrogated by FCM. Specifically, single‐cell suspensions prepared from the spleens were stained with fluorescence‐conjugated antibodies against CD3, CD4, CD8, CD44, and CD62L, whereby T lymphocytes were classified into naive T cells (CD44^−^CD62L^+^), central memory T (Tcm) cells (CD44^+^CD62L^+^), and effector memory T (Tem) cells (CD44^+^CD62L^−^). Encouragingly, the MFS + US treatment markedly promoted the differentiation of memory T lymphocytes in both the CD3^+^CD4^+^ and CD3^+^CD8^+^ compartments, with the proportion of Tcm cells elevating from 8.96% to 19.5% and from 11.5% to 20.3%, respectively, while that of Tem cells simultaneously increasing from 2.36% to 5.49% and from 2.62% to 5.46% (Figure S19). Correspondingly, the fraction of naive T lymphocytes was substantially reduced upon treatment. Notably, Tcm cells possess the capacity for self‐renewal and homing to secondary lymphoid organs, being regarded as the pivotal subset responsible for long‐term immune surveillance and the prevention of tumor metastasis, whereas the concurrent expansion of Tem cells endows the host with rapid effector mobilization upon tumor antigen re‐encounter. Collectively, these findings demonstrate that the combinatory treatment not only elicits a potent anti‐tumor immune response but also establishes durable, memory‐based immunity, thereby providing a favorable immunological basis for the suppression of tumor metastasis.

The robust activation of the anti‐tumor immune system observed in mice has motivated us to conduct an in‐depth exploration of the potential therapeutic mechanisms underlying this process. To comprehensively elucidate these immunological response mechanisms, we employed transcriptomic profiling through RNA sequencing (RNA‐seq) on tumor specimens obtained from the Control group and the MFS + US treatment group at day 7 post‐treatment. Principal component analysis (PCA) was used to evaluate the reproducibility and discriminability between the groups to augment the precision and dependability of subsequent data interpretation (Figure S20). The analysis demonstrated a marked degree of inter‐group differentiation and intra‐group consistency, designating the samples as appropriate subjects for subsequent analytical investigation. Furthermore, volcano plot and heatmap were employed as graphical visualizations to characterize the differentially expressed genes (DEGs) across the two comparative groups (Figure S21 and Figure 8m). Subsequently, a Gene Ontology (GO) enrichment analysis was conducted on the identified DEGs to delineate the immunologically pertinent biological processes in which they exhibit significant enrichment. The bubble diagram revealed that these DEGs are enriched in the biological processes related to antigen processing and presentation, DC maturation, T lymphocyte activation, and immune response (Figure 8n). The GO enrichment results are consistent with the afore‐detected anti‐tumor immune effects, suggesting that following MFS + US treatment in tumor‐bearing mice, there is a significant upregulation of genes associated with anti‐tumor immunity at the tumor tissue. This upregulated gene expression profile promotes the sequential activation of a comprehensive anti‐tumor immune response, ultimately resulting in a pronounced suppression of neoplastic proliferation and metastasis.

## 3 Conclusion and Discussion

Recent studies have elucidated a novel perspective on cancer research by focusing on TRIB as a critical component of tumor tissue, challenging the long‐held assumption of tumor environments as sterile. TRIB has been identified in a variety of cancer types and is implicated in promoting tumor cell proliferation, enhancing invasiveness, and facilitating immune evasion. Notably, convincing evidence suggests a critical role for TRIB in modulating the cytoskeletal dynamics of CTCs, thereby enhancing their resilience and colonization potential. Therefore, the implication of microbial‐infected tumor cells in oncological patients may significantly exceed current recognition, emphasizing the profound clinical significance of TRIB in the carcinogenic process and therapeutic intervention.

Accordingly, we have fully exploited the ROS susceptibility of both tumor cells and intracellular bacteria to design a tumor‐bacteria dual‐killing immunotherapy strategy, highlighting the critical role of TRIB in cancer progression and offering new perspectives and directions for anti‐tumor research. Furthermore, employing CCM coating technology to camouflage the sonosensitizer not only endows the nanosystem with imaging functionalities but also enhances its biocompatibility, thereby facilitating imaging‐guided targeted therapy. Furthermore, the synergistic application of SDT and CDT significantly amplifies the destruction of tumor cells and TRIB, while also triggering a robust systemic immune response, achieving the extensive suppression of the primary neoplasm and a pronounced decrement in the incidence of distant metastases.

Distinct from previous strategies that typically employ distinct pharmacological agents to target tumor cells and intratumoral bacteria [32], the proposed platform integrates these effects via a shared ROS‐mediated mechanism. By leveraging SDT/CDT‐generated ROS as a common trigger, the system induces ferroptotic cell death and bacterial lysis simultaneously, ensuring the temporal alignment of tumor‐associated antigen and bacterial PAMP release within the tumor microenvironment. Crucially, this process is temporally regulated by ultrasound, allowing for on‐demand synchronization of these events. Such coordination creates a highly immunogenic milieu that offers a distinct advantage over passive administration, where the timing of cytotoxic and bactericidal effects is often dissociated. Conventional antibiotic‐based approaches for eliminating TRIB, such as the systemic administration of broad‐spectrum antibiotics [51, 52], frequently suffer from poor tumor penetration, off‐target disruption of the commensal microbiota, and the increasing prevalence of multidrug resistance, whereas our strategy physically eradicates intratumoral bacteria via ROS‐mediated oxidative damage without inducing drug resistance. In contrast to SDT or CDT monotherapies reported previously, whose therapeutic efficacy is often limited by insufficient ROS generation and hypoxia‐associated resistance within the tumor microenvironment [53], the SDT/CDT synergistic system in this study achieves a significantly amplified ROS yield and a superior antitumor outcome. Moreover, unlike bacterial‐based antitumor therapies employing engineered live bacteria [54], which carry inherent risks of uncontrolled proliferation and systemic infection, our CCM‐camouflaged nanosystem demonstrates favorable biosafety and a streamlined preparative procedure. Notably, while most reported nanomedicines target tumor cells alone and leave TRIB intact, thereby allowing residual bacteria to promote recurrence and metastasis [13], our kill‐two‐birds‐with‐one‐stone strategy simultaneously eliminates both tumor cells and TRIB, which may account for the pronounced suppression of distant metastasis observed in this study.

Nevertheless, several limitations of the proposed strategy should be acknowledged. First, the intracellular infection model established in this study serves as a proof‐of‐principle system, in which 4T1 cells are acutely infected with *S. aureus* at a high multiplicity of infection. Such an acute infection may not fully reproduce the characteristics of the low‐abundance, tumor‐adapted intracellular microbiota identified in breast cancer, and future studies employing patient‐derived intracellular bacteria or gnotobiotic models are warranted to validate the physiological relevance of our findings. Second, although the CCM coating confers homologous targeting capability, the heterogeneity of tumor‐associated antigens among patients may compromise the universality of this targeting strategy, and personalized membrane sources or hybrid membrane engineering may be required for clinical translation. Third, the therapeutic efficacy of SDT is inherently dependent on the penetration depth and energy deposition of ultrasound, which may limit its application to deeply seated or irregularly shaped tumors. Fourth, the long‐term biosafety, biodegradation behavior, and potential immunogenicity of the nanosystem have not been fully evaluated in this study, and prolonged pharmacokinetic and toxicity assessments in large animal models are warranted. In addition, the precise composition and functional heterogeneity of TRIB across different tumor types remain to be systematically characterized, which may affect the generalizability of the proposed strategy to other malignancies. Despite these limitations, this study emphasizes the significance of the intratumoral microbiota as a novel target for therapeutic engagement, providing innovative approaches and insights for the clinical treatment of breast cancer. By integrating imaging‐guided targeting, SDT/CDT synergistic therapy, and systemic immune activation into a single nanoplatform, this work offers a safe, broad‐spectrum, and operationally streamlined paradigm for the concurrent eradication of tumor cells and TRIB, and it is anticipated that the therapeutic strategy proposed herein will inspire further research and advancement in the development of treatment modalities for various types of cancers.

## 4 Materials and Methods

### 4.1 Patients and Samples

Cancerous tissues and para‐cancerous tissues (*n* = 8) were collected from TNBC patients with informed consent. The investigation complied with pertinent ethical standards governing human participant research. The collection and use of patient‐derived tumor specimens for research was approved by Affiliated Hospital of Chongqing Medical University (Approval No.: KX2025‐KYC0541‐01).

### 4.2 5R 16S rRNA Gene Sequencing and Bioinformatics Analysis

Total microbial genomic DNA was extracted from the eight paired TNBC tumor and adjacent non‐tumor tissue samples and quantified using a Qubit fluorometer. The V2, V3, V5, V6, and V8 hypervariable regions of the 16S rRNA gene were amplified using a two‐step PCR protocol. The resulting amplicons were purified using AMPure XT beads, quantified, pooled, and subjected to paired‐end sequencing (2 × 150 bp) on the Illumina NovaSeq 6000 platform. Raw sequencing reads were demultiplexed according to their barcode sequences, followed by adapter removal and quality filtering. Taxonomic identification was performed using the Short MUltiple Regions Framework (SMURF), which integrates sequence information from the five amplified regions. Potential contaminants introduced during sample collection, DNA extraction, or PCR amplification were removed based on the negative‐control data. Alpha diversity was evaluated using the observed species, Shannon, Simpson, Chao1, and Good's coverage indices, whereas beta diversity was assessed using Bray‐Curtis distance matrices. Differences in microbial abundance between groups were analyzed using the Mann–Whitney U test, with *p* < 0.05 considered statistically significant.

### 4.3 16S rRNA FISH Analysis

Formalin‐fixed paraffin‐embedded TNBC tissue Sections (4 µm) were hybridized with 16S rRNA probe (EUB338, 5′‐GCTGCCCTCCCGTAGGAGT‐3′) in hybridization buffer. The NON‐EUB338 probe served as a negative control. After washing, nuclei were counterstained with DAPI, and images were acquired by confocal microscopy. Colocalization of 16S signals with tumor cell nuclei was analyzed to determine the intracellular localization of bacteria.

### 4.4 Bio‐TEM Analysis

TNBC tumor tissues were cut into 1 mm^3^ fragments, fixed with 2.5% glutaraldehyde. After dehydration through a graded ethanol series, samples were embedded in epoxy resin. Ultrathin sections were collected on copper grids, double‐stained with uranyl acetate and lead citrate, and examined under a transmission electron microscope to visualize bacterial structures and their localization within tumor cells.

### 4.5 Cell Culture

The murine breast cancer cell line 4T1 (RRID:CVCL_0125) and the murine bone marrow‐derived dendritic cell line DC2.4 (RRID:CVCL_J409) were purchased from Procell Life Science & Technology Co., Ltd. (Wuhan, China). 4T1 cells and DC2.4 cells were cultured in RPMI‐1640 complete medium and incubated at 37°C under a 5% CO_2_ atmosphere.

### 4.6 Intracellular Bacterial Infection Model

A representative methicillin‐resistant Staphylococcus aureus (MRSA) strain, ATCC 43300, was purified and cultured in tryptic soy broth at 37°C for 24 h prior to use. For the establishment of an intracellular infection model, bacteria were harvested via centrifugation and subsequently resuspended in RPMI‐1640 medium supplemented with 10% fetal calf serum. The bacterial suspension was then added to 4T1 cells at a multiplicity of infection of 50 (bacteria per cell) and incubated for 2 h. To eliminate extracellular bacteria, the culture medium was aspirated and replaced with fresh growth medium containing 10 mg L^−1^ lysostaphin, followed by incubation for 1 h. Subsequently, the culture medium was replaced with fresh medium.

### 4.7 Wound‐Healing Assay

*S. aureus*‐harboring 4T1 cells and untreated 4T1 cells were seeded into 6‐well plates and grown to ∼90% confluence, and a uniform scratch was created with a sterile 200 µL pipette tip. After washing with PBS, cells were cultured in serum‐free medium, and images were captured at 0, 24, and 48 h using an inverted phase‐contrast microscope. Wound closure was quantified using ImageJ as: migration rate (%) = (A_0_ − A_t_)/A_0_ × 100%, where A_0_ and A_t_ denote the scratched areas measured at 0 h and 48 h, respectively.

### 4.8 Transwell Migration Assay

Migration was assessed using 24‐well Transwell chambers (8 µm pore, Corning). Cells (5 × 10^4^ in 200 µL serum‐free medium) were seeded into the upper chamber, and 600 µL of complete medium with 10% FBS was added to the lower chamber as a chemoattractant. After 24 h incubation at 37°C with 5% CO_2_, cells on the upper surface were removed with a cotton swab. Migrated cells on the lower surface were fixed with 4% paraformaldehyde, stained with 0.1% crystal violet, photographed, and counted in five randomly selected fields per membrane.

### 4.9 In Vivo Experimental Lung Metastasis Assay

All animal studies and procedures were conducted in accordance with a protocol approved by the Animal Ethics Committee of Chongqing Medical University (Approval No.: IACUC‐CQMU‐2024‐0819). The experiments were performed in strict compliance with the guidelines outlined in the Chongqing Management Approach for Laboratory Animals. Female BALB/c mice (6‐8 weeks old) were obtained from the Experimental Animal Center of Chongqing Medical University. 4T1 cells or *S. aureus*‐harboring 4T1 cells (2 × 10^5^ cells in 100 µL PBS) were injected into the tail vein. Mice were euthanized 14 days later, and the lungs were harvested and fixed in 4% paraformaldehyde. Surface metastatic nodules were photographed and counted in a blinded manner. Lung tissues were paraffin‐embedded, sectioned, and subjected to H&E staining to confirm metastatic lesions.

### 4.10 Materials and Chemicals

Se powder, Polyvinyl pyrrolidone (PVP) and Ferric chloride hexahydrate (FeCl_3_·6H_2_O) were purchased from Adamas Reagent Co., Ltd (Shanghai, China). Sodium borohydride (NaBH_4_) was purchased from Sinopharm Chemical Reagent Co., Ltd (Shanghai, China). 5,5‐Dimethyl‐1‐pyrroline N‐oxide (DMPO) was purchased from Aladdin Biochemical Technology Co., Ltd (Shanghai, China). Reduced Glutathione (GSH) Content Assay Kit, ferrous iron assay kit, Glutathione Peroxidase (GSH‐Px/GPX) Activity Detection Kit, Lipid Peroxidation MDA Assay Kit were purchased from Solarbio Science & Technology Co., Ltd (Beijing, China). ROS Assay Kit, Calcein/PI cell viability and cytotoxicity assay kit, CCK‐8 kits, BCA Protein Assay Kit, 2‐(4‐Amidinophenyl)‐6‐indolecarbamidine dihydrochloride (DAPI) were purchased from Beyotime Technology Co., Ltd (Shanghai, China). BODIPY ^581/591^ C11 was purchased from Thermo Fisher Scientific Inc. (Waltham, MA, USA). FITC fluorescent antibodies against *S. aureus* were purchased from Abcam PLC (Cambridge, UK). Enzyme‐linked immunosorbent assay (ELISA) kits including mouse interleukin‐6 (IL‐6), tumor necrosis factor‐α (TNF‐α), and interferon‐γ (IFN‐γ) were purchased from Meimian Industrial Co., Ltd (Jiangsu, China).

### 4.11 Synthesis of FS and MFS NSs

The initial FS nanosheets (NSs) were synthesized via a simple stepwise hydrothermal method. Specifically, 40 mL of deionized water was purged with nitrogen for 30 min to remove dissolved oxygen. Subsequently, Se powder (79 mg) and NaBH_4_ (76 mg) were introduced, and the mixture was stirred until a clear and transparent solution was obtained. Then, PVP (200 mg) and FeCl_3_·6H_2_O (146 mg) were added sequentially with continuous stirring, resulting in an immediate color change of the solution to black. The above solution is then transferred to a 50 mL Teflon‐lined autoclave and subjected to a hydrothermal reaction at 220°C for 12 h. After the reaction, the product is washed and finally preserved by freeze‐drying.

To obtain FS NSs with enhanced stability and biocompatibility, 10 mg of initial FS NSs were dispersed in 10 mL of deionized water. Subsequently, an excess of NH2‐mPEG was introduced, and the resultant mixture was subjected to ultrasonication for 30 min under ice bath conditions. Then, the mixture was washed and centrifuged multiple times, and the obtained FS NSs were re‐suspended in deionized water for further use.

For the preparation of MFS NSs, a mixture of FS NSs (2 mg) and 4T1 tumor cell membranes (0.5 mg) in 1 mL of deionized water was subjected to ultrasonication for 5 min under ice‐cooled conditions. Subsequent centrifugation and washing steps were employed to remove any unbound cell membrane components.

### 4.12 Biocompatibility Evaluation of MFS NSs

Healthy BALB/c mice (6‐8 weeks old) were randomly divided into five groups (*n* = 5), representing various time points post‐intravenous injection: 0, 3, 7, 14, and 30 days. Following intravenous administration of MFS NSs (2 mg mL^−^^1^, 200 µL), blood samples and major organs (heart, liver, spleen, lung, and kidney) were harvested. The blood samples were collected for routine hematological parameters and serum biochemical indices. Specifically, liver function was assessed via markers including alanine aminotransferase (ALT), aspartate transaminase (AST), and total bilirubin (TBIL). Renal function was evaluated using creatinine (CREA) and urea nitrogen (BUN) levels, while myocardial integrity was gauged by measuring the myocardial enzyme profile, comprising creatine kinase (CK) and L‐lactate dehydrogenase (LDH). Subsequently, the major organs were processed for histological examination using H&E staining.

### 4.13 Instruments and Characterization

The morphology was observed by scanning electron microscopy (SEM, Hitachi SU‐9000) and transmission electron microscopy (TEM, JEOL JEM‐2100F). The x‐ray diffraction (XRD) analysis was performed by Rigaku D/MAX‐2200 PC XRD system. The Raman analysis was conducted on a LABRAM HR Evolution Lab‐HRDLS 20 spectrometer. X‐ray photoelectron spectroscopy (XPS) measurements were performed using Thermo ESCALAB 250. The x‐ray absorption near‐edge structures (XANES) analysis was performed on the Shanghai Synchrotron Radiation Facility (SSRF). The UV–vis–DRS and UV–vis–NIR absorption spectra were recorded by a U‐3600 spectrometer (Shimadzu, Japan). Theoretical calculations were performed using the density functional theory (DFT) approach, implemented within the Vienna Ab initio Simulation Package (VASP). To confirm the successful encapsulation of the cell membrane on the surface of FS NSs, we conducted Sodium Dodecyl Sulfate‐Polyacrylamide Gel Electrophoresis (SDS‐PAGE) followed by Coomassie Blue staining. Size distribution and zeta potential were characterized using a dynamic light scattering analyzer (Opptronix 919SZ, Shanghai, China).

### 4.14 US‐Triggered Catalytic Performance of FS NSs

The generation of ^1^O_2_ triggered by ultrasound (US) irradiation in FS NSs was evaluated by monitoring the degradation of 1,3‐diphenylisobenzofuran (DPBF) using UV–Vis–NIR spectroscopy. Specifically, FS NSs (100 µg mL^−^^1^) dispersed in 2 mL phosphate buffer (PBS) were mixed with 2 mL of ethanol containing DPBF (80 µg mL^−^^1^).  Then, the mixture was mixed thoroughly in the dark conditions before being irradiated with ultrasound (1 MHz, 1 W cm^−2^, 50% duty cycle) for designated time durations: 0, 1, 2, 4, and 8 min. Following irradiation, the reaction solution was centrifuged to pellet the FS NSs. The resulting supernatant was analyzed using UV–Vis–NIR spectroscopy to record absorbance spectra between 350 and 550 nm. Ultrasound‐treated DPBF samples served as control groups. The decrease in absorbance at 410 nm, corresponding to the degradation of DPBF, was measured to quantify the generation of ^1^O_2_.

### 4.15 Fenton‐Mediated ·OH Production of FS NSs

To evaluate the hydroxyl radical (·OH) production mediated by the Fenton reaction, the colorimetric oxidation of 3,3',5,5'‐tetramethylbenzidine (TMB) was employed as an indicator. The reaction system, comprising TMB (1 mg mL^−^^1^), NaHCO_3_ (25 mM), H_2_O_2_ (2 mM), and various concentration of FS NSs (0, 50, 100, 200 µg mL^−^^1^), was dissolved in PBS (pH 6.5). The solution was then reacted under dark conditions for 30 min to allow for radical generation and subsequent TMB oxidation. Following this incubation period, FS NSs were removed via centrifugation to isolate the supernatant. Finally, the resulting supernatant was visually inspected for color changes and further analyzed using UV−Vis−NIR spectroscopy to obtain the absorbance spectrum within the 300–800 nm range.

To further validate the generation of ·OH via the Fenton reaction, electron spin resonance (ESR) spectroscopy was employed usng the DMPO‐trapping agent. A dispersion of FS NSs (100 µg mL^−^^1^) in water containing H_2_O_2_ (2 mM) was thoroughly mixed with DMPO (5 mM). The presence of ·OH radicals was subsequently detected via ESR analysis. For comparative purposes, control experiments were conducted using mixtures of FS + DMPO, H_2_O_2_ + DMPO, and H_2_O + DMPO under identical conditions.

### 4.16 GSH Depletion of FS NSs

The GSH depletion capacity of FS NSs was detected using Reduced Glutathione (GSH) Content Assay Kit. Briefly, GSH (350 µg mL^−^^1^) was incubated with varying concentrations of FS NSs (0, 50, 100, 200 µg mL^−^^1^) in PBS (pH 6.5). The reaction mixtures were incubated for predetermined time intervals (0, 5, 10, 15, 20, 25, 30 min), after which the remaining GSH concentration was quantified according to the manufacturer's protocol.

### 4.17 Intracellular Uptake

The intracellular uptake of FS and MFS NSs by 4T1 cells was investigated using confocal laser scanning microscopy (CLSM, Olympus, Japan). 4T1 cells were seeded at a density of 1 × 10^5^ cells per well in confocal‐specific dishes and incubated for 24 h. Subsequently, the culture medium was replaced with fresh medium containing FITC‐labeled FS or MFS NSs, respectively. Following incubation periods of 0, 2, 4, 6, 8, and 10 h, cells were washed twice with PBS and fixed with 4% paraformaldehyde for 15 min. Finally, prior to CLSM imaging, cellular nuclei were stained with DAPI to facilitate cell identification and localization of the internalized NSs. Furthermore, the intracellular fluorescence intensity of FITC‐labeled NSs was also quantitively analyzed via flow cytometry (FCM, LSR Fortessa, BD Biosciences).

### 4.18 Detection of Intracellular ROS and LPO

The intracellular ROS levels were analyzed using a DCFH‐DA probe. 4T1 cells (1 × 10^5^) were seeded in confocal‐specific dishes and randomly assigned into 6 groups including: (1) Control, (2) US, (3) FS, (4) MF, (5) FS + US, (6) MFS + US. The cells were co‐incubated with various NSs (NSs concentration: 100 µg mL^−1^) for 8 h. Subsequently, the culture medium was replaced with fresh medium. Cells in group 2, 5 and 6 were then exposed to US irradiation (1 MHz, 1 W cm^−2^, 50% duty cycle) for 2 min. Following irradiation, the cells were returned to the incubator for further culture. After an additional 4 h, the cells were gently washed three times with PBS. They were then incubated with the DCF‐DA probe at 37°C for 20 min in the dark conditions. After another gentle PBS wash (three times), intracellular fluorescence was visualized and imaged using a CLSM. Furthermore, the intracellular ROS fluorescence intensity was also quantitatively determined by FCM assay. For the intracellular lipid peroxidation (LPO) assay, treated cells were incubated with the BODIPY ^581/591^ C11 fluorescent probe (10 µM) at 37°C for 30 min. Subsequently, fluorescence was also visualized and imaged using CLSM.

### 4.19 In Vitro Cytotoxicity Assay

To evaluate the cytotoxicity of MFS NSs, 4T1 cells (1 × 10^5^) were seeded in confocal‐specific dishes for 24 h and then subjected to the previously described grouping (1) Control, (2) US, (3) FS, (4) MF, (5) FS + US, (6) MFS + US) and treatments. Subsequently, the cells were stained using a Calcein‐AM and PI solution, allowing the visual differentiation of living and dead cells via CLSM. Specifically, live cells were stained bright green fluorescence by Calcein‐AM, whereas dead cells were stained red fluorescence by PI. In addition, the CCK‐8 assay was utilized for a further assessment of the therapeutic effect of the synthesized material. In brief, 4T1 cells (5 × 10^3^) were seeded into 96‐well plates for 24 h. Thereafter, the cells were subjected to the aforementioned grouping and treatments. And the cell viability was then measured using CCK‐8 kits.

### 4.20 Evaluation of Fe^2+^ Content

4T1 cells (1 × 10^5^) were seeded in 6‐well plates and incubated for 24 h. Subsequently, the culture medium was replaced with fresh medium containing the following groups: (1) Control, (2) FS, (3) MF. After an 8‐h incubation, the cells were gently washed multiple times with PBS, harvested, and then assayed for ferrous iron content according to the manufacturer's instructions provided with the ferrous iron assay kit.

### 4.21 Intracellular GSH Depletion, GPX Activity and MDA Content Assay

Following 24‐h adhesion of 4T1 cells (2 × 10^6^ cells) seeded in the cell culture dishes, the cultures were divided into 6 experimental groups: (1) Control, (2) US, (3) FS, (4) MF, (5) FS + US, (6) MFS + US. Following an 8‐h incubation period, the cells were gently washed with PBS and replenished with fresh culture medium. Subsequently, groups 2, 5, and 6 were subjected to US irradiation (1 MHz, 1 W cm^−2^, 50% duty cycle) for 2 min. After a further 4 h of incubation, the cells were washed twice with PBS and harvested for the quantification of GSH content using a Reduced GSH Content Detection Kit. 4T1 cells cultured in 6‐well plates and subjected to the aforementioned grouping and treatments were subsequently evaluated for GPX activity via a Glutathione Peroxidase (GSH‐Px/GPX) Activity Detection Kit. In parallel, Western blot analysis (WB) was conducted to further assess the impact of the various treatments on GPX expression levels. The intracellular concentration of malondialdehyde (MDA) was measured using a Lipid Peroxidation MDA Assay Kit. Moreover, protein levels in the cellular samples were assessed employing a BCA Protein Assay Kit.

### 4.22 Mitochondrial Analysis

Following the aforementioned grouping and treatments, 4T1 cells cultured in 6‐well plates were stained with a Mitochondrial Membrane Potential Assay Kit containing JC‐1, and their mitochondrial membrane potential was assessed by FCM assay. In order to directly visualize the impact of the treatment on mitochondrial structure, the cells were collected, fixed with 3% glutaraldehyde, and subsequently examined for mitochondrial morphology using TEM.

### 4.23 Intracellular Antimicrobial Assay

*S. aureus*‐harboring 4T1 cells were randomly divided into the following experimental groups: (1) Control, (2) US, (3) FS, (4) MF, (5) FS + US, and (6) MF + US. Following an 8‐h incubation period, the cells were gently washed with phosphate‐buffered saline (PBS) and replenished with fresh culture medium. Thereafter, groups 2, 5, and 6 were subjected to ultrasound irradiation (1 MHz, 1 W cm^−2^, 50% duty cycle) for 2 min. Finally, cells were cultured for an additional 24 h.

To quantify intracellular bacterial load, infected cells were lysed by incubation with 1 mL sterile water for 10 min. Then, the lysates were serially diluted, and aliquots of appropriate dilutions were plated onto Sheep Blood Agar plates for colony‐forming units (CFUs) enumeration after incubation at 37°C. Additionally, the intracellular bacterial clearance efficiency was further evaluated using FCM assay. Infected cells were fixed (4% paraformaldehyde), permeabilized (0.2% Triton‐X100), blocked (1% BSA), and stained with FITC‐labeled anti‐*S. aureus* antibody at 4°C for 30 min. Following three times washes with PBS, the FITC fluorescence in cells were analyzed by FCM examination.

To visualize intracellular *S. aureus*, treated cells were analyzed by CLSM and Bio‐TEM. For CLSM imaging, subsequent to the aforementioned processing steps, the cellular cytoskeleton was stained with Phalloidin‐TRITC, followed by nuclear counterstaining with DAPI. Subsequently, fluorescence signals were acquired and imaged using CLSM. For Bio‐TEM observation, infected cells treated with NSs and US were centrifuged and fixed in 2.5% glutaraldehyde. Subsequently, the cells were washed with 0.1 M sodium cacodylate buffer, followed by postfixation with 1% osmium tetroxide and being dehydrated through a graded ethanol series (30%, 50%, 70%, 80%, 90%, 95% and 100%) for 10 min at each gradient. After staining with 2% uranyl acetate and infiltration with Epon resin, ultrathin sections were prepared and imaged using Bio‐TEM (Hitachi HT‐7800, Japan).

### 4.24 In Vitro DC Maturation

DC maturation was evaluated in vitro using a Transwell system. 4T1 cells (without *S. aureus* infection) subjected to 6 distinct treatments (Control, US, FS, MF, FS + US, MFS + US) were seeded into the upper compartment, while DC2.4 cells were seeded into the lower compartment. Following 24 h of incubation, DC2.4 morphology was assessed and imaged using an optical microscope. Subsequently, DC2.4 cells were harvested and stained with fluorescently labeled antibodies against CD11c, CD80, and CD86. Maturation status was analyzed via FCM. Furthermore, the levels of proinflammatory cytokines IL‐6 and TNF‐α secreted by DC2.4 cells were quantified in the culture supernatant using ELISA kits.

To dissect the respective contributions of bacteria‐derived PAMPs and tumor cell death‐derived DAMPs in the complete system, a Transwell system was employed with five distinct treatments: Control, *S. aureus* + MFS + US, 4T1 + MFS + US, S. aureus‐harboring 4T1 + antibiotic + MFS + US, and *S. aureus*‐harboring 4T1 + MFS + US. *S. aureus*‐harboring 4T1 cells were prepared as described above. Briefly, for *S. aureus* + MFS + US group, *S. aureus* was co‐incubated with MFS NSs under US irradiation, and the treated bacteria were seeded into the upper compartment. For the 4T1‐related groups, *S. aureus*‐free 4T1 cells or *S. aureus*‐harboring 4T1 cells were treated with MFS NSs + US, with or without linezolid pretreatment to eliminate the TRIB, and subsequently seeded into the upper compartment. DC2.4 cells were seeded into the lower compartment and co‐incubated for 24 h. Thereafter, DC2.4 cells were harvested and stained with fluorescently labeled antibodies against CD11c, CD80, and CD86, and the DC maturation levels were analyzed via FCM.

### 4.25 4T1 Tumor‐Bearing Mice Models

The 4T1 breast cancer animal model was established by subcutaneously injecting 1 × 10^6^ logarithmically growing 4T1 cells, resuspended in 50 µL serum‐free medium, into the subcutaneous tissue of the right mammary fat pad.

### 4.26 Biodistribution and Tumor Penetration of MFS NSs

The biodistribution of MFS NSs in a 4T1 tumor‐bearing mouse model was characterized using the in vivo fluorescence imaging system (IVIS Lumina II, PerkinElmer, USA). Mice received an intravenous (i.v.) injection of Cy5.5‐labeled FS or MFS NSs (2 mg mL^−^^1^, 200 µL). Fluorescence signals were acquired and recorded at 0, 2, 4, 8, 12, and 24 h postinjection using the imaging system. Additionally, representative tumor tissues from two groups were collected and prepared for immunofluorescence staining for further analysis.

To evaluate tumor penetration, the accumulation of FS or MFS NSs within tumor tissue was evaluated using the Vevo LAZR Photoacoustic Imaging System (Visual Sonics Inc., Toronto, Canada). In vitro photoacoustic (PA) imaging was performed on FS NSs suspensions across a range of concentrations (62.5, 125, 250, 500, 1000 µg mL^−^^1^). Quantified PA signal intensities were analyzed using the accompanying Vevo LAZR software. For in vivo studies, PA imaging was conducted at designated time points before and after (2, 4, 8, 12, and 24 h) intravenous administration of FS or MFS NSs (2 mg mL^−^^1^, 200 µL), and the resulting PA signal intensities within the tumor regions were measured.

### 4.27 Antitumor Efficacy of US‐Triggered MFS NSs

To evaluate the effects of US‐triggered MFS NSs on tumor growth inhibition, 4T1 tumor‐bearing mice (*n* = 5) were randomly divided into 6 groups: (1) Control, (2) US, (3) FS, (4) MF, (5) FS + US, (6) MFS + US. According to the grouping, mice were administered intravenously with saline, FS or MFS NSs. Subsequently, groups 2, 5, and 6 received US irradiation (1 MHz, 2 W cm^−2^, 50% duty cycle, 2 min) at 6, 8, and 10 h postinjection, respectively. To monitor treatment efficacy, tumor dimensions and mice body weights were measured every other day post‐treatment. Tumor volume was calculated as length × (width)^2^ × 0.5. At day 20, mice were euthanized, and tumors were excised for weighing and imaging. Additionally, a random tumor tissue from each group was subjected to further analysis, including immunofluorescence for CD4 and CD8, standard histological processing (H&E staining), TUNEL assay for apoptosis detection, and PCNA staining to assess proliferative activity.

### 4.28 Metastasis Inhibition

To quantitatively evaluate the efficacy of metastasis inhibition, the presence and extent of metastatic nodules within lung tissue were assessed. In brief, lung tissues were harvested from euthanized mice and subjected to fixation in Bouin's solution for 30 min. Subsequently, macroscopically visible metastatic nodules on the lung surfaces were documented via imaging. Furthermore, representative lung tissue sections from each experimental group were processed for H&E staining to provide a detailed microscopic analysis.

### 4.29 In Vivo Immune Activation

To assess the in vivo DC maturation, experimental cohorts were subjected to identical treatment protocols as those employed in the antitumor efficacy studies. 3 days subsequent to US irradiation, tumor tissues, sentinel lymph nodes (SLNs), and spleens were harvested. These tissues were subsequently processed for FCM analysis, involving surface staining with antibodies conjugated to CD11c‐FITC, CD80‐APC, and CD86‐PE. Concurrently, murine serum samples were collected to quantify the levels of pro‐inflammatory cytokines, specifically IL‐6 and TNF‐α.

To evaluate T cell infiltration, 4T1 tumor‐bearing mice were administered the identical treatment regimen. 7 days postirradiation, lymphocytes isolated from tumor tissues and spleens were stained with corresponding fluorophore‐conjugated antibodies. FCM analysis was then performed to characterize the infiltration of CD3^+^CD8^+^ T cells and CD3^+^CD4^+^Foxp3^+^ T cells. Furthermore, the production of IFN‐γ was assessed using corresponding ELISA kits.

### 4.30 Statistical Analysis

Data are expressed as mean ± s.d. and n represents biologically independent experiments. Statistical analysis was performed using a two‐tailed Student's *t*‐test for two independent groups and a one‐way or two‐way ANOVA for three or more groups (ns represent not significant, ^*^ *p* < 0.05, ^**^ *p* < 0.01, ^***^ *p* < 0.001).

## Author Contributions

Conceptualization: L.Z., H.L., M.G., X.L. Methodology: X.L., M.G., H.L., L.Z., T.J., Z.G. Investigation: X.L., L.Z., M.G., T.J., Z.G., Z.Z., Y.C. Formal analysis: M.G., H.L., L.Z. Data curation: X.L., M.G. Visualization: X.L., M.G. Writing – original draft: X.L., M.G. Writing – review & editing: M.G., H.L., L.Z. Supervision: H.L., L.Z., P.L.

## Funding

The National Natural Science Foundation of China 82202175 (L.Z.), 22422510 (H.L.), 52372276 (H.L.).

## Conflicts of Interest

The authors declare no conflicts of interest.

## Supporting information




**Supporting File**: advs78559‐sup‐0001‐SuppMat.docx.

## Data Availability

The data that supports the findings of this study are available in the supplementary material of this article.

## References

[1] L. S. Popovic , G. Matovina‐Brko , M. Popovic , K. Punie , A. Cvetanovic , and M. Lambertini , “Targeting Triple‐Negative Breast Cancer: A Clinical Perspective,” Oncology Research 31, no. 3 (2023): 221–238, 10.32604/or.2023.028525. PMC10229315 37305385

[2] G. Bergers and S.‐M. Fendt , “The Metabolism of Cancer Cells During Metastasis,” Nature Reviews Cancer 21, no. 3 (2021): 162–180, 10.1038/s41568-020-00320-2. PMC8733955 33462499

[3] G. Follain , D. Herrmann , S. Harlepp , et al., “Fluids and Their Mechanics in Tumour Transit: Shaping Metastasis,” Nature Reviews Cancer 20, no. 2 (2020): 107–124, 10.1038/s41568-019-0221-x. 31780785

[4] A. Fu , B. Yao , T. Dong , and S. Cai , “Emerging Roles of Intratumor Microbiota in Cancer Metastasis,” Trends in Cell Biology 33, no. 7 (2023): 583–593, 10.1016/j.tcb.2022.11.007. 36522234

[5] D. R. Welch and D. R. Hurst , “Defining the Hallmarks of Metastasis,” Cancer Research 79, no. 12 (2019): 3011–3027, 10.1158/0008-5472.CAN-19-0458. PMC6571042 31053634

[6] T. Hou , X. Huang , J. Lai , and D. Zhou , “Intra‐Tumoral Bacteria in Breast Cancer and Intervention Strategies,” Advanced Drug Delivery Reviews 217 (2025): 115516, 10.1016/j.addr.2025.115516. 39828126

[7] Y. Huang , M. Su , Y. Zhang , et al., “Microbiota‐Induced EI24 Improves Homeostasis but Impedes Function of Alveolar Macrophages via Metabolic Regulation,” Nature Communications 17, no. 1 (2026): 2227, 10.1038/s41467-026-69000-3. PMC12963607 41620436

[8] W. Guo , Y. Zhang , S. Guo , et al., “Tumor Microbiome Contributes to an Aggressive Phenotype in the Basal‐Like Subtype of Pancreatic Cancer,” Communications Biology 4, no. 1 (2021): 1019, 10.1038/s42003-021-02557-5. PMC8408135 34465850

[9] D. Nejman , I. Livyatan , G. Fuks , et al., “The Human Tumor Microbiome is Composed of Tumor Type–Specific Intracellular Bacteria,” Science 368, no. 6494 (2020): 973–980, 10.1126/science.aay9189. PMC7757858 32467386

[10] L. Yang , A. Li , Y. Wang , and Y. Zhang , “Intratumoral Microbiota: Roles in Cancer Initiation, Development and Therapeutic Efficacy,” Signal Transduction and Targeted Therapy 8, no. 1 (2023): 35, 10.1038/s41392-022-01304-4. PMC9842669 36646684

[11] Y. Li , S. Xing , F. Chen , et al., “Intracellular Fusobacterium Nucleatum Infection Attenuates Antitumor Immunity in Esophageal Squamous Cell Carcinoma,” Nature Communications 14, no. 1 (2023): 5788, 10.1038/s41467-023-40987-3. PMC10507087 37723150

[12] B. A. Helmink , M. A. Wadud Khan , A. Hermann , V. Gopalakrishnan , and J. A. Wargo , “The Microbiome, Cancer, and Cancer Therapy,” Nature Medicine 25, no. 3 (2019): 377–388, 10.1038/s41591-019-0377-7. 30842679

[13] A. Fu , B. Yao , T. Dong , et al., “Tumor‐Resident Intracellular Microbiota Promotes Metastatic Colonization in Breast Cancer,” Cell 185, no. 8 (2022): 1356–1372.e26, 10.1016/j.cell.2022.02.027. 35395179

[14] A. G. Lopez , “Microbial Strategist of Metastasis,” Nature Reviews Microbiology 20, no. 6 (2022): 318, 10.1038/s41579-022-00738-5. 35418615

[15] X. An , W. Yu , J. Liu , D. Tang , L. Yang , and X. Chen , “Oxidative Cell Death in Cancer: Mechanisms and Therapeutic Opportunities,” Cell Death & Disease 15, no. 8 (2024): 556, 10.1038/s41419-024-06939-5. PMC11294602 39090114

[16] H. Sies , V. V. Belousov , N. S. Chandel , et al., “Defining Roles of Specific Reactive Oxygen Species (ROS) in Cell Biology and Physiology,” Nature Reviews Molecular Cell Biology 23, no. 7 (2022): 499–515, 10.1038/s41580-022-00456-z. 35190722

[17] B. Perillo , M. Di Donato , A. Pezone , et al., “ROS in Cancer Therapy: The Bright Side of the Moon,” Experimental & Molecular Medicine 52, no. 2 (2020): 192–203, 10.1038/s12276-020-0384-2. PMC7062874 32060354

[18] H. Zhai , L. Lv , H. Ren , et al., “Multimodal Antibacterial Agents Based on Photodynamic Therapy and Photothermal Therapy: Principles, Design Strategies and Applications,” Chemical Society Reviews 55, no. 16 (2026): 8653–8700, 10.1039/D6CS00072J. 42453065

[19] X. Jia , E. Wang , and J. Wang , “Rational Design of Nanozymes for Engineered Cascade Catalytic Cancer Therapy,” Chemical Reviews 125, no. 5 (2025): 2908–2952, 10.1021/acs.chemrev.4c00882. 39869790

[20] K. M. Nowak , M. R. Schwartz , V. R. Breza , and R. J. Price , “Sonodynamic Therapy: Rapid Progress and New Opportunities for Non‐Invasive Tumor Cell Killing With Sound,” Cancer Letters 532 (2022): 215592, 10.1016/j.canlet.2022.215592. PMC8918024 35151824

[21] Y. Zhang , X. Zhang , H. Yang , et al., “Advanced Biotechnology‐Assisted Precise Sonodynamic Therapy,” Chemical Society Reviews 50, no. 20 (2021): 11227–11248, 10.1039/D1CS00403D. 34661214

[22] Z. Jiang , W. Xiao , and Q. Fu , “Stimuli Responsive Nanosonosensitizers for Sonodynamic Therapy,” Journal of Controlled Release 361 (2023): 547–567, 10.1016/j.jconrel.2023.08.003. 37567504

[23] L. Yan , L. Chang , Y. Tian , et al., “Graphene Quantum Dot Sensitized Heterojunctions Induce Tumor‐Specific Cuproptosis to Boost Sonodynamic and Chemodynamic Enhanced Cancer Immunotherapy,” Advanced Science 12 (2025): 2410606, 10.1002/advs.202410606. PMC11831527 39716968

[24] X. Pan , Z. Huang , J. Guo , et al., “MOF‐Derived Nanoparticles With Enhanced Acoustical Performance for Efficient Mechano‐Sonodynamic Therapy,” Advanced Materials 36, no. 33 (2024): 2400142, 10.1002/adma.202400142. 38896775

[25] Y. Nie , W. Chen , Y. Kang , et al., “Two‐Dimensional Porous Vermiculite‐Based Nanocatalysts for Synergetic Catalytic Therapy,” Biomaterials 295 (2023): 122031, 10.1016/j.biomaterials.2023.122031. 36731367

[26] S. George , S. Pokhrel , Z. Ji , et al., “Role of Fe Doping in Tuning the Band Gap of TiO_2_ for the Photo‐Oxidation‐Induced Cytotoxicity Paradigm,” Journal of the American Chemical Society 133, no. 29 (2011): 11270–11278, 10.1021/ja202836s. PMC3971840 21678906

[27] Y. Li , W. Chen , Y. Kang , et al., “Nanosensitizer‐Mediated Augmentation of Sonodynamic Therapy Efficacy and Antitumor Immunity,” Nature Communications 14, no. 1 (2023): 6973, 10.1038/s41467-023-42509-7. PMC10620173 37914681

[28] G. Liu , N. Ma , K. Cheng , et al., “Bacteria‐Derived Nanovesicles Enhance Tumour Vaccination by Trained Immunity,” Nature Nanotechnology 19, no. 3 (2024): 387–398, 10.1038/s41565-023-01553-6. 38052943

[29] J. Zheng , F. Zhao , E. Pariente , et al., “Tumor‐Targeted Glutamine Metabolism Blocker Synergizes With TiO_2_‐Au Janus Nanoparticles for Enhanced Sono‐Metabolic Antitumor Therapy,” Advanced Materials 37, no. 12 (2025): 2418800, 10.1002/adma.202418800. 39950402

[30] H. Sun , W. Hu , Y. Yan , et al., “Using PAMPs and DAMPs as Adjuvants in Cancer Vaccines,” Human Vaccines & Immunotherapeutics 17, no. 12 (2021): 5546–5557, 10.1080/21645515.2021.1964316. PMC8903972 34520322

[31] B. Ding , P. Zheng , J. Tan , et al., “Sodium Bicarbonate Nanoparticles for Amplified Cancer Immunotherapy by Inducing Pyroptosis and Regulating Lactic Acid Metabolism,” Angewandte Chemie International Edition 62, no. 40 (2023): 202307706, 10.1002/anie.202307706. 37587061

[32] X. Liu , M. Sun , F. Pu , J. Ren , and X. Qu , “Transforming Intratumor Bacteria Into Immunopotentiators to Reverse Cold Tumors for Enhanced Immuno‐Chemodynamic Therapy of Triple‐Negative Breast Cancer,” Journal of the American Chemical Society 145, no. 48 (2023): 26296–26307, 10.1021/jacs.3c09472. 37987621

[33] S. Liang , B. Liu , X. Xiao , et al., “A Robust Narrow Bandgap Vanadium Tetrasulfide Sonosensitizer Optimized by Charge Separation Engineering for Enhanced Sonodynamic Cancer Therapy,” Advanced Materials 33, no. 36 (2021): 2101467, 10.1002/adma.202101467. 34296464

[34] R. H. Fang , W. Gao , and L. Zhang , “Targeting Drugs to Tumours Using Cell Membrane‐Coated Nanoparticles,” Nature Reviews Clinical Oncology 20, no. 1 (2023): 33–48, 10.1038/s41571-022-00699-x. 36307534

[35] T. Cui , S. Chen , S. Liu , et al., “A Biohybrid Chiral Hydrogel Enhances Preclinical Postoperative Glioblastoma Therapy by Multi‐Pronged Inhibition of Tumour Stemness,” Nature Nanotechnology 21, no. 1 (2026): 140–155, 10.1038/s41565-025-02064-2. 41454130

[36] M. Yu , Z. Ye , S. Liu , et al., “Redox‐Active Ferrocene Quencher‐Based Supramolecular Nanomedicine for NIR‐II Fluorescence‐Monitored Chemodynamic Therapy,” Angewandte Chemie International Edition 63 (2024): 202318155, 10.1002/anie.202318155. 38109458

[37] Y. Chen , J. Zhang , H. Liu , and Z. Wang , “Controlled Synthesis of FeSe_2_ Nanoflakes toward Advanced Sodium Storage Behavior Integrated With Ether‐Based Electrolyte,” Nano 13, no. 12 (2018): 1850141, 10.1142/S1793292018501412.

[38] C. Ji , F. Liu , L. Xu , and S. Yang , “Urchin‐Like NiCo_2_O_4_ Hollow Microspheres and FeSe_2_ Micro‐Snowflakes for Flexible Solid‐State Asymmetric Supercapacitors,” Journal of Materials Chemistry A 5, no. 11 (2017): 5568–5576, 10.1039/C6TA11001K.

[39] H. Y. Jeong , J. H. Lee , and K. F. Hayes , “Characterization of Synthetic Nanocrystalline Mackinawite: Crystal Structure, Particle Size, and Specific Surface Area,” Geochimica et Cosmochimica Acta 72, no. 2 (2008): 493–505, 10.1016/j.gca.2007.11.008. PMC2981034 21085620

[40] X. Wang , N. Zhang , H. Shang , et al., “Precisely Designing Asymmetrical Selenium‐Based Dual‐Atom Sites for Efficient Oxygen Reduction,” Nature Communications 16, no. 1 (2025): 470, 10.1038/s41467-025-55862-6. PMC11707329 39775107

[41] S. Jiang , M. Xiang , J. Zhang , et al., “Rational Design of Hierarchical FeSe_2_ Encapsulated With Bifunctional Carbon Cuboids as an Advanced Anode for Sodium‐Ion Batteries,” Nanoscale 12, no. 43 (2020): 22210–22216, 10.1039/D0NR06359B. 33140808

[42] S.‐S. Mai , K.‐Y. Hsiao , Y.‐C. Yang , et al., “Synchronous Regulation of Schottky/P‐N Dual Junction in Prussian Blue‐Derived Janus Heterostructures: A Path to Ultrafast Long Life Potassium Ion Batteries,” Chemical Engineering Journal 474 (2023): 145992, 10.1016/j.cej.2023.145992.

[43] G. Li , S. Wu , J. Liu , K. Wang , X. Chen , and H. Liu , “Narrow Bandgap Schottky Heterojunction Sonosensitizer With High Electron–Hole Separation Boosted Sonodynamic Therapy in Bladder Cancer,” Advanced Materials 36, no. 26 (2024): 2401252, 10.1002/adma.202401252. 38549283

[44] M. Ma , Y. Zhang , K. Pu , and W. Tang , “Nanomaterial‐Enabled Metabolic Reprogramming Strategies for Boosting Antitumor Immunity,” Chemical Society Reviews 54, no. 2 (2025): 653–714, 10.1039/D4CS00679H. 39620588

[45] J. Zhu , C. Sevencan , M. Zhang , et al., “Increasing the Potential Interacting Area of Nanomedicine Enhances Its Homotypic Cancer Targeting Efficacy,” ACS Nano 14, no. 3 (2020): 3259–3271, 10.1021/acsnano.9b08798. 32049490

[46] Q. Wang , Y. Tian , M. Yao , J. Fu , L. Wang , and Y. Zhu , “Bimetallic Organic Frameworks of High Piezovoltage for Sono‐Piezo Dynamic Therapy,” Advanced Materials 35, no. 41 (2023): 2301784, 10.1002/adma.202301784. 37432882

[47] X. Xue , M. Wang , J. Cui , et al., “Glutathione Metabolism in Ferroptosis and Cancer Therapy,” Cancer Letters 621 (2025): 217697, 10.1016/j.canlet.2025.217697. 40189013

[48] Y. Zhu , P. Gong , J. Wang , et al., “Amplification of Lipid Peroxidation by Regulating Cell Membrane Unsaturation to Enhance Chemodynamic Therapy,” Angewandte Chemie International Edition 62, no. 12 (2023): 202218407, 10.1002/anie.202218407. 36708200

[49] H. Hu , S. Hua , F. Lu , et al., “Mucous Permeable Nanoparticle for Inducing Cuproptosis‐Like Death in Broad‐Spectrum Bacteria for Nebulized Treatment of Acute Pneumonia,” Advanced Science 12, no. 15 (2025): 2408580, 10.1002/advs.202408580. PMC12005761 39985298

[50] Q. Hu , Y. Zhang , H. Lou , et al., “GPX4 and Vitamin E Cooperatively Protect Hematopoietic Stem and Progenitor Cells From Lipid Peroxidation and Ferroptosis,” Cell Death & Disease 12, no. 7 (2021): 706, 10.1038/s41419-021-04008-9. PMC8282880 34267193

[51] C. K. Stein‐Thoeringer , N. Y. Saini , E. Zamir , et al., “A Non‐Antibiotic‐Disrupted Gut Microbiome Is Associated With Clinical Responses to CD19‐CAR‐T Cell Cancer Immunotherapy,” Nature Medicine 29, no. 4 (2023): 906–916, 10.1038/s41591-023-02234-6. PMC10121864 36914893

[52] L. M. Chambers , E. L. Esakov Rhoades , R. Bharti , et al., “Disruption of the Gut Microbiota Confers Cisplatin Resistance in Epithelial Ovarian Cancer,” Cancer Research 82, no. 24 (2022): 4654–4669, 10.1158/0008-5472.CAN-22-0455. PMC9772178 36206317

[53] J. Li , Z. Yue , M. Tang , et al., “Strategies to Reverse Hypoxic Tumor Microenvironment for Enhanced Sonodynamic Therapy,” Advanced Healthcare Materials 13, no. 1 (2024): 2302028, 10.1002/adhm.202302028. 37672732

[54] V. Raman , C. P. Deshpande , S. Khanduja , L. M. Howell , N. Van Dessel , and N. S. Forbes , “Build‐a‐Bug Workshop: Using Microbial‐Host Interactions and Synthetic Biology Tools to Create Cancer Therapies,” Cell Host & Microbe 31, no. 10 (2023): 1574–1592, 10.1016/j.chom.2023.09.006. 37827116

