# Supplementary material for: Nanocatalytic Immunotherapy Targeting Tumor‐Resident Intracellular Bacteria for Triple‐Negative Breast Cancer Treatment and Metastasis Suppression
Source: Adv Sci (Weinh). 2026 Oct 31:e78559. Online ahead of print. doi: 10.1002/advs.78559 (PMC13723710; doi:10.1002/advs.78559)
Supplement: Supplementary file 1 [file ADVS-9999-e78559-s001.docx]

Supporting Information

Nanocatalytic immunotherapy targeting tumor-resident intracellular bacteria for triple-negative breast cancer treatment and metastasis suppression

Xiaojing Leng^1§^, Tao Jiang^2§^, Zhao Guo^3§^, Zhimin Zhang^4^, Yihan Chen^4^, Pan Li^1^, Min Ge^5*^, Han Lin^4*^, and Liang Zhang^1*^

1. Department of Ultrasound, The First Affiliated Hospital of Chongqing Medical University, 1 Youyi Road, Yuzhong District, Chongqing 400016, P. R. China.

2. Department of Anaesthesiology, Chongqing Traditional Chinese Medicine Hospital, 6 Panxi Qizhi Road, Jiangbei District, Chongqing, 400021, China.

3. Department of Orthopedic Surgery, Shanghai Institute of Microsurgery on Extremities, Shanghai Sixth People's Hospital Affiliated to Shanghai Jiao Tong University School of Medicine, 600 Yishan Road, Shanghai, 200233, China

4. Shanghai Institute of Ceramics Chinese Academy of Sciences; Research Unit of Nanocatalytic Medicine in Specific Therapy for Serious Disease, Chinese Academy of Medical Sciences, Shanghai, 200050, China.

5. Department of Electrical Engineering, City University of Hong Kong, Tat Chee Avenue, HKSAR, 999077, China.

^§^These authors contributed equally to this work.

*Corresponding author: Min Ge: gmin@mail.ustc.edu.cn. Han Lin: linhan@mail.sic.ac.cn.

Liang Zhang: zhangliang338@cqmu.edu.cn.

**This PDF file includes:**

Supporting figure S1 to S21

**Supporting figures**

**Figure S1. Intracellular bacteria in human TNBC specimens.** (a) 16S rRNA FISH staining on cancerous sections of TNBC patients. Green signals represent 16S rRNA-positive bacteria (white arrows). (b) Bio-TEM images of cancerous tissues from TNBC patients, showing bacteria-like structures within the cytosol of tumor cells (white arrows).

**Figure S2. Intracellular *S. aureus* colonization enhances the migratory capacity of 4T1 breast cancer cells.** (a) Representative scratch wound healing assay images of 4T1 cells and *S. aureus*-harboring 4T1 cells at 0, 24, and 48 h post-scratching (Yellow lines outline the wound boundary). (b) Quantification of migration rate at 48 h (n = 5). (c) Representative images of crystal violet-stained 4T1 cells that migrated through Transwell membranes. (d) Quantification of migrated cell numbers per microscopic field (n = 15).

**Figure S3. TRIB promote metastatic colonization of tumor cells *in vivo*.** (a) Representative digital photos of excised lung tissue in 4T1 group and *S. aureus*-harboring 4T1 cells group. (b) Number of visible tumor nodules in lung tissues between groups (n = 5). (c) Representative images of H&E-stained lung tissues.

**Figure S4.** (a) The photographs of PEGylated FS nanosheets (FS-PEG) and initial FS nanosheets (FS). (b) The polydispersity index (PDI) data of FS-PEG and FS (n = 5).

**Figure S5.** (a) Time dependent oxidation of DPBF under US irradiation (1 MHz, 1 Wcm^−2^, 50% duty cycle). (b) Comparison of DPBF oxidation by US only and FS + US at 410 nm.

**Figure S6.** (a) Hematological parameters and biochemical markers of the mice after intravenous administration of MFS nanosheets at different time points (0, 3, 7, 14, and 30 d) (n = 5). (b) Representative photomicrographs of H&E-stained sections from major organs of mice subjected to various treatments (scale bar: 100 μm).

**Figure S7.** Representative CLSM images of JC-1 dye-stained 4T1 cells after various treatments (scale bar: 50 μm).

**Figure S8.** Cell viabilities of 4T1 cells treated by MFS + US regimen and varied concentrations of ferroptosis inhibitors (a) EDTA, (b) ferrostatin-1, (c) GSH, and (d) vitamin E (n = 5).

**Figure S9.** Cell viabilities of 4T1 cells treated by MFS + US regimen and varied concentrations of cell death pathways inhibitors (a) Z-VAD-FMK, (b) 3-methyladenine, and (c) necrostatin-1 (n = 5).

**Figure S10. Intracellular co-localization of MFS with *S. aureus* in 4T1 cells.** (a) CLSM images of tumor cells incubated with FITC-labeled MFS nanosheets for 0.5, 3, and 12 h. Lysosomes and nuclei were stained with LysoTracker (red) and DAPI (blue), respectively. (b) Representative CLSM images showing the physical contact between DiI-labeled MFS (red) and FITC-labeled *S. aureus* (green) in 4T1 cells. Nuclei were stained with DAPI (blue).

**Figure S11.** (a) The translocation of CRT after various treatments (scale bar: 50 μm). (b) The release of HMGB-1 after various treatments (scale bar: 50 μm).

**Figure S12.** The expression levels of IL-6 secreted from DC2.4 cells co-incubating with 4T1 cells after various treatments (n = 5).

**Figure S13. DC maturation induced by bacteria-derived PAMPs and tumor cell death-derived DAMPs.** (a) Representative FCM analysis plots and (b) relative quantification of DC maturation (gated at CD11c^+^ CD80^+^ CD86^+^) (n = 5).

**Figure S14.** PA signal intensity value of MFS nanosheets within the excitation wavelength range of 680 nm-790 nm.

**Figure S15.** *In vitro* analysis PA images and PA intensities of MFS nanosheets at varying concentrations (n = 3).

**Figure S16.** (a) *In vivo* PA imaging of tumors post-intravenous injection with FS and MFS nanosheets (scale bar: 1 mm) and (b) corresponding quantitative analysis of PA intensities within tumor regions (n = 3).

**Figure S17.** (a) Fluorescence images of 4T1 tumor-bearing mice at various timepoints (pre-injection, 2, 4, 8 and 24 h). (b) Frozen section micrographs of tumor tissue obtained 8 h following injection.

**Figure S18.** Body weight variations across the therapeutic intervention period (n = 5).

**Figure S19. Analysis of memory T cell subsets in the spleen after different treatments.** (a) Representative flow cytometric plots of CD44 and CD62L expression in splenic CD3⁺CD4⁺ and CD3⁺CD8⁺ T cells, whereby the cells were classified into naive T cells (CD44⁻CD62L⁺), Tcm (CD44⁺CD62L⁺), and Tem (CD44⁺CD62L⁻). (b-c) Quantification of the proportions of naive, Tcm, and Tem subsets (n = 5).

**Figure S20.** PCA biplot with 95% confidence intervals for sample clustering in the Control and MFS + US groups (n = 3).

**Figure S21.** Volcano plots illustrating DEGs in the in the Control and MFS + US groups. Volcano plots depicting the differentially expressed genes (DEGs) between the Control and MFS + US groups (n = 3; fold change > 1.5, pValue < 0.05).
